# GLTSCR1 Negatively Regulates BRD4‐Dependent Transcription Elongation and Inhibits CRC Metastasis

**DOI:** 10.1002/advs.201901114

**Published:** 2019-10-16

**Authors:** Fengyan Han, Lei Zhang, Chaoyi Chen, Yan Wang, Yi Zhang, Lili Qian, Wenjie Sun, Dan Zhou, Beibei Yang, Honghe Zhang, Maode Lai

**Affiliations:** ^1^ Department of Pathology Key Laboratory of Disease Proteomics of Zhejiang Province Research unit of intelligence classification of tumor pathology and precision therapy Chinese Academy of Medical Sciences (2019RU042) School of Medicine Zhejiang University Hangzhou 310058 China; ^2^ Department of Pharmacology China Pharmaceutical University Nanjing 210009 China

**Keywords:** frameshift mutations, glioma tumor suppressor candidate region gene 1 (GLTSCR1), microsatellite instability (MSI), transcription elongation

## Abstract

Frameshift mutations frequently occur in colorectal cancer (CRC) with microsatellite instability (MSI), but the nature and biological function of many MSI‐associated mutations remain elusive. Here, an MSI frameshift mutation is identified in glioma tumor suppressor candidate region gene 1 (*GLTSCR1*) that produces two C‐terminal‐truncated proteins. Additionally, *GLTSCR1* is verified as a tumor suppressor that inhibits CRC metastasis. Through binding to bromodomains and the phosphorylation‐dependent interaction domain of bromodomain protein 4 (BRD4) via the C‐terminus, GLTSCR1 blocks oncogenic transcriptional elongation. However, truncated GLTSCR1 translocates into the cytoplasm and loses BRD4 binding domain, which induces the phosphorylation of RNA Pol II at Ser2 and dephosphorylation at Ser5, then increases oncogenic transcriptional elongation. Importantly, GLTSCR1 deficiency decreases sensitivity to bromodomain and extra terminal domain inhibitors. This study highlights the molecular mechanism of the GLTSCR1‐BRD4 interaction, which is a potential therapeutic target for CRC.

## Graphical abstract

This study reports a microsatellite instability‐selected frameshift mutation in glioma tumor suppressor candidate region gene 1 (*GLTSCR1*) that converts *GLTSCR1* from an antimetastatic gene to a prometastatic gene. A bromodomain protein 4‐GLTSCR1‐DNA model is proposed in which the truncated GLTSCR1 coregulates the oncogenic transcriptional elongation and these findings provide a strong rationale for targeting GLTSCR1 in colorectal cancer.

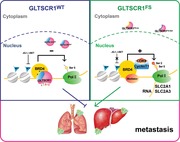

## 1 Introduction

Mismatch repair (MMR) is an important cellular process maintaining fidelity in DNA replication through correcting mismatched DNA sequences.^1^ A defective MMR system causes a mutational phenotype leading to a predisposition to cancer.^2^ As a molecular marker of a deficient MMR system, microsatellite instability (MSI) may lead to the production of truncated protein products and result in oncogenic potential when it occurs in coding regions of genes involved in several crucial functions and pathways.^3^, ^4^ MSI not only represents a molecular hallmark of hereditary nonpolyposis Lynch syndrome but also occurs in ≈15–20% of sporadic colorectal cancer (CRC) cases.^5^ In addition to CRC, MSI has also been observed in endometrial cancer, ovarian cancer, clear cell renal cell carcinoma, etc.^6^ Accumulating evidence suggests that MSI can predict a more favorable clinical prognosis and an effective response to chemotherapy and immunotherapy.^7^ Insertion/deletion (indel) mutations in the microsatellite sequence of target genes might be positively selected during tumor development and progression. Therefore, frameshift mutations usually accumulate in these repeated sequences of target genes in cancers with a high frequency of MSI (MSI‐H), resulting in the loss of function of key genes, and could be considered as diagnostic or therapeutic targets.^3^, ^4^ Although some microsatellite sites have been elucidated in detail, screening and identification of additional novel functional microsatellite sites are essential for understanding CRC development.

Chromosome 19 not only has the highest gene density among all human chromosomes but also carries a high density of repeat sequences. Nearly 55% of this chromosome consists of repetitive elements.^8^ Located at 19q13.33, *GLTSCR1* was reported as a glioma tumor suppressor candidate region gene because allelic loss of the chromosome 19q arm is a frequent event in human diffuse gliomas.^9^ GLTSCR1 is ubiquitously expressed in spleen, prostate, adipose, and colon tissues and participates in the formation of the mammalian SWI/SNF chromatin remodeling complexes to regulate gene expression and genome integrity.^10^ Polymorphisms of *GLTSCR1* are associated not only with the development and progression of oligodendroglioma but also with the aggressiveness of lung cancer.^11^ In addition, the expression of *GLTSCR1* is associated with the progression of prostate cancer.^12^ However, the clinical significance of *GLTSCR1* expression in other solid tumors such as CRC remains unknown. Moreover, the molecular mechanism by which GLTSCR1 contributes to human development and disease is poorly understood. Although liquid chromatography‐tandem mass spectrometry studies in HEK293 cells identified GLTSCR1 as a novel bromodomain protein 4 (BRD4)‐interacting protein, which performed a positive transcription elongation factor b (pTEFb)‐independent transcriptional activation function,^13^ the model of interaction between GLTSCR1 and BRD4 remains to be clarified, and the importance and biological roles of this complex in various diseases, including cancer, await definition.

In this study, we identified a new microsatellite site in *GLTSCR1* that caused a frameshift mutation and produced a truncated GLTSCR1 protein in MSI‐H CRC. Furthermore, GLTSCR1 played an antimetastatic role through interacting with BRD4 to regulate the transcriptional elongation of target genes. More importantly, GLTSCR1 deficiency decreased sensitivity to bromodomain and extra terminal domain (BET) inhibitors, which exhibit therapeutic activity in many types of cancer.^14^ Finally, MSI mutation or expression of *GLTSCR1* could be considered as a biomarker of BET inhibitor response for precision therapy in CRC.

## 2 Results

### 2.1 *GLTSCR1* DNA C8 Microsatellite Site Frameshift Mutations Occur in MSI‐H CRC

**Figure 1:**
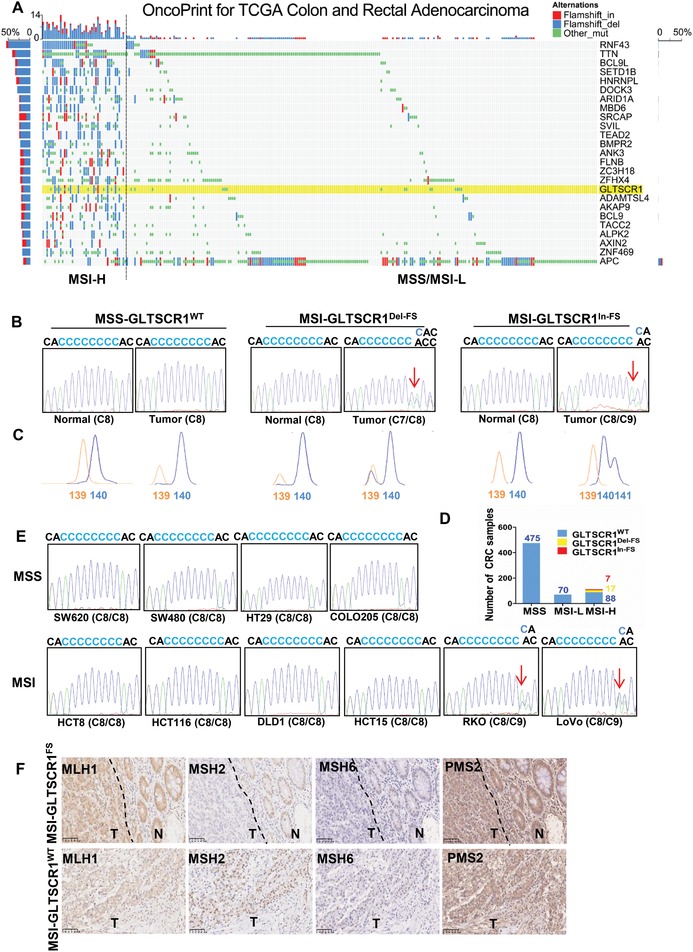
Frameshift mutation in the *GLTSCR1* C8 microsatellite site in CRC. A) Frameshift mutation profile of CRC samples from the TCGA database (*GLTSCR1* is highlighted in yellow). B) Representative DNA sequences of the *GLTSCR1* C8 microsatellite site in paired normal and CRC samples (the arrows indicate the mutation site). C) Fluorescence capillary electrophoresis of the *GLTSCR1* C8 microsatellite site in paired normal and CRC samples (the orange peak is the 139 bp marker on the DNA ladder; the wild‐type *GLTSCR1* PCR product is 140 bp). D) Distribution of the *GLTSCR1* C8 mutation in CRC samples with different MSI status (*n* = 657). E) DNA sequence of the *GLTSCR1* C8 microsatellite site in CRC cell lines (the arrows indicate the mutation site). F) Representative images of standardized immunostaining for four MMR proteins in CRC samples (N, normal tissue; T, tumor tissue; the dashed line represents the boundary between the normal and tumor tissue).

MSI‐H cancers exhibit a typical spectrum of mutations that distinguish them from microsatellite‐stable (MSS)/MSI‐low (MSI‐L) cancers.^15^ To investigate the specific frameshift mutations in MSI‐H CRC, we reanalyzed CRC data in the Cancer Genome Atlas (TCGA) database. The frameshift mutation frequency in the MSI‐H subgroup was much higher than that in the MSS/MSI‐L subgroup and these frameshift mutations in MSI‐H CRC were discovered more frequently in the tandem repeat sequences of tumor suppressor genes. (**Figure** **1**A and Table S1, Supporting Information). Among these mutations, an eight‐cytosine (C8) mononucleotide short tandem repeat in the sixth exon of the *GLTSCR1* gene was identified as a novel microsatellite site, at which the indel mutation (C9/C7) resulted in a *GLTSCR1* frameshift mutation in CRC samples (highlighted in Figure 1A and Table S1, Supporting Information).

Next, we assessed the MSI status of 657 sporadic CRC samples and paired normal tissues from our tissue bank by using five National Cancer Institute (NCI)‐recommended MSI markers: BAT25, BAT26, D5S346, D17S250, and D2S123 (Figure S1A, Supporting Information). A total of 112 (17%, 112/657) CRC samples were MSI‐H, 70 (11%, 70/657) were MSI‐L, and 475 (72%, 475/657) were MSS (Figure S1B, Supporting Information). Then, polymerase chain reaction (PCR) amplification combined with DNA sequencing was used to further verify heterozygous frameshift mutations with the deletion or insertion of a single cytosine nucleotide in the *GLTSCR1* C8 microsatellite site in these CRC samples (Figure 1B); these results were reconfirmed by capillary electrophoresis (Figure 1C). Interestingly, two types of mutations in the C8 microsatellite site of *GLTSCR1* were found in only 24 (21.4%, 24/112) of the MSI‐H CRC samples, but no mutations were observed in any MSI‐L or MSS CRC samples (Figure 1D). Among the 24 samples with mutations, 17 samples exhibited the deletion mutation (*GLTSCR1^Del‐FS^* in Figure 1D), and seven exhibited the insertion mutation (*GLTSCR1^In‐FS^* in Figure 1D). In addition, we detected this mutation in all CRC cell lines in our laboratory, including six lines with MSI (HCT8, HCT116, DLD1, HCT15, RKO, and LOVO) and four MSS lines (SW620, SW480, HT29, and COLO205). As expected, the heterozygous frameshift mutation occurred only in the RKO and LOVO MSI cell lines (Figure 1E). To assess whether these mutations were caused by deficiency of the MMR system, we performed an immunochemistry assay to investigate the expression of four MMR proteins (MLH1, MSH2, MSH6, and PMS2) and found that these mutations were usually associated with downregulated expression of MSH2 and MSH6 (Figure 1F). Taken together, these results indicate a novel microsatellite site in the *GLTSCR1* gene, at which the indel frameshift alteration is an MSI‐H‐specific mutation in CRC.

### 2.2 GLTSCR1 Inhibits Tumor Metastasis in CRC

**Figure 2:**
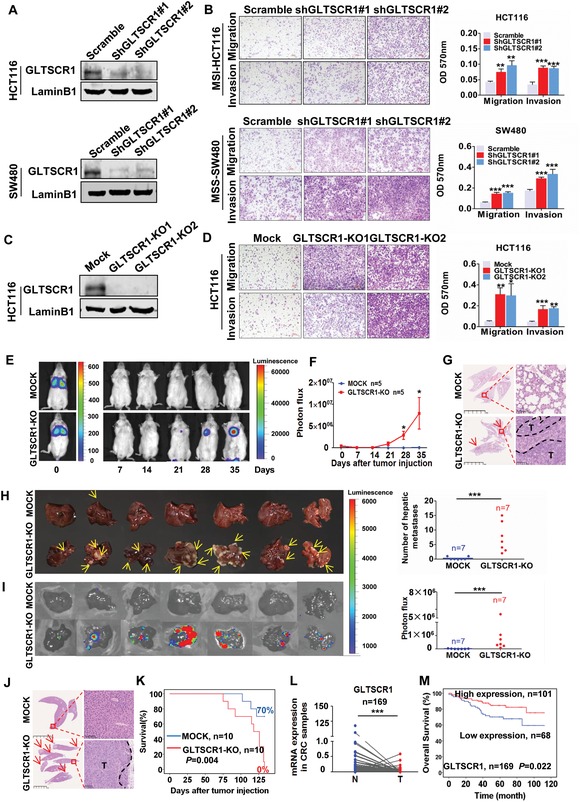
GLTSCR1 inhibits CRC metastasis both in vitro and in vivo. A) Immunoblotting analysis of GLTSCR1 protein expression in HCT116 (upper) and SW480 (lower) cells transfected with scrambled shRNA and shGLTSCR1. B) Transwell assay to investigate the migratory and invasive properties of HCT116 (upper) and SW480 (lower) cells transfected with scrambled shRNA and shGLTSCR1. The histograms on the right show the quantification analysis results. C) Immunoblotting analysis of GLTSCR1 protein expression in control (mock) and GLTSCR1‐KO (GLTSCR1‐KO1 and KO2) HCT116 cells. D) Transwell assay to investigate the migratory and invasive properties of control (mock) and GLTSCR1‐KO (GLTSCR1‐KO1 and KO2) HCT116 cells. The histogram on the right shows the quantification analysis results. E) Representative bioluminescence images of the lung metastasis model established by tail vein injection of control (mock) and GLTSCR1‐KO HCT116 cells in NOD/SCID mice. F) Quantification of the photon flux in pulmonary metastatic luciferase foci. Data are presented as the mean ± standard deviation (SD); statistical significance was assessed by an unpaired *t*‐test. *P < 0.05, *n* = 5. G) H&E staining in pulmonary metastatic foci (E). The arrows indicate metastatic foci; T, tumor tissue. H) Images of the liver distant metastasis model established by splenic injection of control (mock) and GLTSCR1‐KO HCT116 cells in BALB/c nu/nu mice (the yellow arrows indicate metastatic foci). The right scatter plot shows the quantification analysis results. I) Bioluminescence images of the liver metastasis model (H). The right scatter plot shows the quantification analysis of the photon flux in pulmonary metastatic foci. Data are presented as the mean ± SD; statistical significance was assessed by the Mann–Whitney *U*‐test. *P < 0.05, *n* = 7. J) H&E staining in pulmonary metastatic foci (H). The arrows indicate metastatic foci; T, tumor tissue. K) Kaplan–Meier plots of the overall survival of mice in the splenic injection liver metastasis model. *P* = 0.004, *n* = 10. L) Relative mRNA expression of *GLTSCR1* in paired normal and tumor samples from our tissue bank. Four of the data points are outside the axis limits. Data are presented as the mean ± SD; statistical significance was assessed by a paired *t*‐test. ***P < 0.001, *n* = 169. M) Kaplan–Meier plots of the overall survival of CRC patients. *P* = 0.022. Data are presented as the mean ± SD; statistical significance was assessed by an unpaired *t*‐test. *P < 0.05, *P < 0.05, **P < 0.01, ***P < 0.001; *n* = 3.

Although *GLTSCR1* has been defined as a glioma tumor suppressor candidate region gene for two decades,^9^ the biological function of GLTSCR1 in tumorigenesis remains unknown. To determine the biological roles of wild‐type GLTSCR1 (GLTSCR1^WT^) in CRC, we assessed GLTSCR1 expression in the HCT116, SW480, SW620, DLD1, HCT8, RKO, and LOVO cell lines by RT‐PCR and immunoblotting, which showed that HCT116 and SW480 cells have relatively high GLTSCR1^WT^ expression (Figure S2A, Supporting Information). Then, GLTSCR1 expression was knocked down in HCT116 and SW480 cells by shRNA (**Figure** **2**A and Figure S2B, Supporting Information). Knockdown of GLTSCR1^WT^ expression increased the migration and invasion potential in both cell lines (Figure 2B). To further verify the function of GLTSCR1 in CRC cells, we knocked out GLTSCR1^WT^ in HCT116 cells by using sgRNA‐clustered regularly interspaced short palindromic repeats (CRISPR)/Cas9 (Figure 2C and Figure S2C, Supporting Information). Deletion of GLTSCR1^WT^ promoted CRC cell migration and invasion, consistent with the effects of GLTSCR1 knockdown in HCT116 cells (Figure 2D). But GLTSCR1 knockout (GLTSCR1‐KO) had no effect on cell proliferation (Figure S2D,E, Supporting Information). These results indicate that GLTSCR1^WT^ could inhibit CRC cell migration and invasion in vitro.

We next investigated whether GLTSCR1 could suppress CRC metastasis in vivo. Stably transfected luciferase‐labeled control (mock) or GLTSCR1‐KO HCT116 cells (Figure S2F, Supporting Information) were injected intravenously into non‐obese diabetes/severe combined immunodeficiency (NOD/SCID) mice, which were then subjected to bioluminescence imaging (BLI). The GLTSCR1‐KO group exhibited visible metastatic luciferase foci on the 28th day, but no signal was observed in the control group until the 35th day (Figure 2E), suggesting that GLTSCR1 inhibited the extravasation and/or early seeding of lung metastatic cells. During continued BLI monitoring, GLTSCR1‐KO cells exhibited continued metastatic outgrowth in the lungs of mice (Figure 2F). The numbers and sizes of the metastatic lesions were drastically increased in GLTSCR1‐KO group mice, as evidenced by hematoxylin and eosin (H&E) staining (Figure 2G). In addition, mock and GLTSCR1‐KO HCT116 cells with stable luciferase expression were orthotopically inoculated into the spleen of BALB/c nu/nu nude mice via surgery to establish the liver distant metastasis model. Consistent with the results in the lung metastasis model, GLTSCR1‐KO group mice exhibited a significantly increased number of metastatic foci in the liver (Figure 2H) and significant enhancement of the fluorescence signals (Figure 2I). H&E staining showed that more and larger metastatic foci were present in the liver of GLTSCR1‐KO group mice than in the liver of control mice (Figure 2J). More importantly, GLTSCR1 knockout significantly shortened the survival time of mice compared with that of control mice. During the 125 d period, the survival of mice inoculated with GLTSCR1‐KO cells dropped by 70% with respect to that of control mice (Figure 2K).

To determine the clinical roles of GLTSCR1 in CRC, we assessed *GLTSCR1* mRNA levels in 169 CRC and paired normal tissues by real‐time quantitative polymerase chain reaction (RT‐qPCR). As shown in Figure 2L, *GLTSCR1* expression was significantly downregulated in CRC samples (Figure 2L). Prognostic analysis showed that GLTSCR1_high patients had a significantly increased overall survival time compared with that of GLTSCR1_low patients (Figure 2M). With the above data, these findings indicate that *GLTSCR1* acts as an antimetastatic gene in CRC development and serves as a biomarker for more favorable prognosis.

### 2.3 Frameshift Mutations Lead to Loss of GLTSCR1 Function

**Figure 3:**
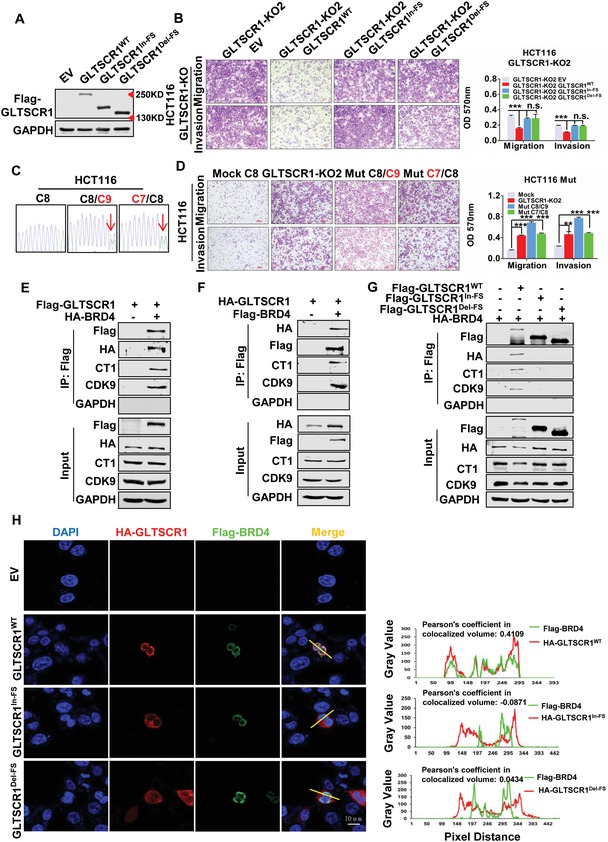
Truncated GLTSCR1^FS^ loses its antimetastatic function. A) Immunoblotting analysis of GLTSCR1 protein expression in GLTSCR1‐KO HCT116 cells with re‐expression of Flag‐GLTSCR1^WT^, GLTSCR1^Del‐FS^, and GLTSCR1^In‐FS^. B) Transwell assay to investigate the migration and invasion potential of GLTSCR1‐KO HCT116 cells with re‐expression of Flag‐GLTSCR1^WT^, GLTSCR1^Del‐FS^, and GLTSCR1^In‐FS^. The histogram on the right shows the quantification analysis results. C) DNA sequence of the GLTSCR1 C8 microsatellite site in CRISPR‐mediated heterozygous‐mutated HCT116 cell lines (the arrows indicate the mutation site). D) Transwell assay to investigate the migration and invasion potential of control (mock) and CRISPR‐mediated heterozygous‐mutated HCT116 cell lines (GLTSCR1‐KO2 for positive control). The histogram on the right shows the quantification analysis results. E) Immunoblotting to detect the immunoprecipitation of exogenous Flag‐tagged GLTSCR1, HA‐tagged BRD4, CT1 and CK9 by an anti‐Flag antibody in HEK293 cells. F) Immunoblotting to detect the immunoprecipitation of exogenous Flag‐tagged BRD4, HA‐tagged GLTSCR1, CT1 and CK9 by an anti‐Flag antibody in HEK293 cells. G) Immunoblotting to detect the immunoprecipitation of exogenous Flag‐tagged GLTSCR1^WT^, GLTSCR1^Del‐FS^ or GLTSCR1^In‐FS^, HA‐tagged BRD4, CT1 and CK9 by an anti‐Flag antibody in HEK293 cells. H) Immunofluorescence staining for exogenous Flag‐tagged BRD4 and HA‐tagged GLTSCR1^WT^, GLTSCR1^Del‐FS^ or GLTSCR1^In‐FS^ in HCT116 cells. Nuclei were stained with DAPI. Scale bars, 10 µm. The *Y*‐axis represents measurements of fluorescent intensity and the *X*‐axis represents the drawn distances for colocalization analysis (the Pearson's coefficient in colocalized volume for the colocalization index). Data are presented as the mean ± SD; statistical significance was assessed by an unpaired *t*‐test. *P < 0.05, *P < 0.05, **P < 0.01, ***P < 0.001; *n* = 3.

Previous data showed indel frameshift mutations in the *GLTSCR1* C8 microsatellite site, which is located in the sixth exon and spanned codons 935–937 (the poly‐Pro domain). According to the prediction, both insertion and deletion frameshift mutations of the *GLTSCR1* C8 site would result in a premature stop codon and the production of two types of truncated GLTSCR1 proteins (referred to as GLTSCR1^FS^; GLTSCR1^In‐FS^, and GLTSCR1^Del‐FS^) (Figure S3A, Supporting Information). As most frameshift mutations in tumor suppressor genes result in a loss of function,^3^, ^16^ we investigated the functional effect of GLTSCR1^FS^ in CRC. Flag‐tagged GLTSCR1^WT^, GLTSCR1^Del‐FS^, and GLTSCR1^In‐FS^ expression vectors were constructed and separately transfected into GLTSCR1‐KO HCT116 cells. Immunoblotting showed that these vectors successfully re‐expressed the wild‐type and the two types of truncated GLTSCR1 proteins in GLTSCR1‐KO HCT116 cells (**Figure** **3**A). Interestingly, as evidenced by Transwell assay, re‐expression of GLTSCR1^WT^ in GLTSCR1‐KO cells reduced the potential for migration and invasion, which was increased by GLTSCR1 knockout, while re‐expression of GLTSCR1^Del‐FS^ and GLTSCR1^In‐FS^ did not significantly affect migration and invasion (Figure 3B). To mimic human pathology, we generated the heterozygous MSI‐associated GLTSCR1^FS^ mutated HCT116 cell lines through CRISPR/Cas9 (Figure S3B, Supporting Information, and Figure 3C) and examined the biological effects of GLTSCR1 indel mutation, which significantly promoted cell migration and invasion (Figure 3D). Then we also corrected the heterozygous insertion mutation (C9) of mutated‐RKO cell line (Figure S3B,C, Supporting Information) and the potential of migration and invasion went into reverse (Figure S3D, Supporting Information). These results demonstrated that both insertion and deletion frameshift mutations in the C8 site result in a loss of GLTSCR1 function, which abolished the inhibition of CRC cell migration and invasion.

A previous study reported an interaction between GLTSCR1 and BRD4 by mass spectrometry analysis.^13^ To probe whether indel frameshift mutations affect the interaction between GLTSCR1 and BRD4, we coexpressed HA‐labeled BRD4 (HA‐BRD4) and Flag‐labeled GLTSCR1 (Flag‐GLTSCR1) in HEK293 cells. A coimmunoprecipitation (co‐IP) assay with an anti‐Flag antibody showed that GLTSCR1 could pull down not only BRD4 but also CDK9 and CT1 (Figure 3E), which are the members of the pTEFb protein complex.^17^ Similarly, BRD4 coimmunoprecipitated with GLTSCR1, as it did with CDK9 and CT1 (Figure 3F). And the endogenous interaction between BRD4 and GLTSCR1 was also confirmed by co‐IP (Figure S3E, Supporting Information). Another co‐IP assay with BRD4 and the GLTSCR1^FS^ expression vectors revealed that neither GLTSCR1^Del‐FS^ nor GLTSCR1^In‐FS^ could bind to BRD4 or the pTEFb complex (Figure 3G). Furthermore, the endogenous interaction was immediately abolished in heterozygous MSI‐associated GLTSCR1^FS^ mutated HCT116 (Figure S3F, Supporting Information). BRD4 executes its function on transcriptional regulation in the nucleus,^18^ and GLTSCR1 contains a nuclear localization sequence (NLS) at amino acids (aa) 1468–1479 (GLPPAKRRKSES) to import it into nuclear. However, the truncated GLTSCR1^FS^ protein, which should be translocated to the cytoplasm, lost its C‐terminus downstream from aa 937. As expected, immunofluorescence assays showed that GLTSCR1^WT^ and BRD4 colocalized in the nucleus, but GLTSCR1^Del‐FS^ and GLTSCR1^In‐FS^ were exported from the nucleus and localized in the cytoplasm (Figure 3H and Figure S3G, Supporting Information). Collectively, these data suggest that the carboxy (C)‐terminal truncation of GLTSCR1^FS^ results in its translocation into the cytoplasm, loss of its antimetastatic function, and defective bind to intranuclear BRD4.

### 2.4 GLTSCR1 Interacts with BRD4 through the C‐Terminal Domain (CTD) to Inhibit CRC Migration and Invasion

**Figure 4:**
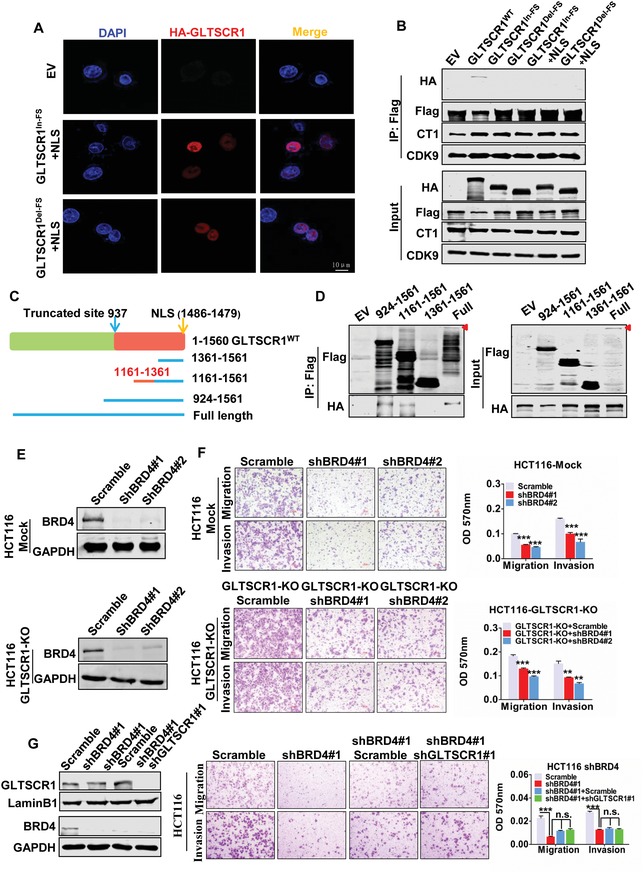
The C‐terminus of GLTSCR1 regulates CRC metastasis through interacting with BRD4. A) Immunofluorescence staining for HA‐GLTSCR1^Del‐FS^ + NLS or HA‐GLTSCR1^In‐FS^ + NLS in HCT116 cells. Nuclei were stained with DAPI. Scale bars, 10 µm. B) Immunoblotting to detect the immunoprecipitation of exogenous Flag‐tagged BRD4, HA‐tagged HA‐GLTSCR1^WT^ HA‐GLTSCR1^Del‐FS^, HA‐GLTSCR1^In‐FS^, HA‐GLTSCR1^Del‐FS^ + NLS, and HA‐GLTSCR1^In‐FS^ + NLS by an anti‐Flag antibody in HEK293 cells. C) Schematic of the C‐terminus of GLTSCR1 and the GLTSCR1 deletion constructs. D) Immunoblotting to detect the immunoprecipitation of exogenous Flag‐tagged GLTSCR1 with different deletion fragments and HA‐tagged BRD4 by an anti‐Flag antibody in HEK293 cells. E) Immunoblotting analysis of BRD4 protein expression in scrambled shRNA and shBRD4‐transfected HCT116 mock (upper panel) and HCT116 GLTSCR1‐KO (lower panel) cells. F) Transwell assay for investigating migration and invasion potential in scrambled shRNA‐ and shBRD4‐transfected HCT116 mock (upper panel) and HCT116 GLTSCR1‐KO (lower panel) cells. The histograms on the right show the quantification analysis results. G) Immunoblotting analysis of BRD4 and GLTSCR1 protein expression in HCT116 cells transfected with shBRD4 and shBRD4 + shGLTSCR1 (left panel); Transwell assay to investigate the migration and invasion potential (middle panel) in these cells. The histograms on the right show the quantification analysis results. Data are presented as the mean ± SD; statistical significance was assessed by an unpaired *t*‐test. *P < 0.05, **P < 0.01, ***P < 0.001; *n* = 3.

To determine which domains of GLTSCR1 are important for binding to BRD4 and whether GLTSCR1^Del‐FS^ and GLTSCR1^In‐FS^ do not interact with BRD4 merely due to their translocation. We cloned the NLS into GLTSCR1^Del‐FS^ and GLTSCR1^In‐FS^ expression vectors. The NLS assisted the nuclear reentry of both GLTSCR1 truncation mutants, as demonstrated by the immunofluorescence assay (**Figure** **4**A). However, co‐IP assays showed that even nuclear GLTSCR1^Del‐FS^ and GLTSCR1^In‐FS^ could not interact with BRD4 and the pTEFb complex (Figure 4B). These results suggest that the C‐terminus (downstream of aa 937) of GLTSCR1 is the key domain for BRD4 binding and that this important region is deleted in GLTSCR1^FS^. Next, we generated several C‐terminal mutation constructs of GLTSCR1 (Figure 4C) and coexpressed them with BRD4 in HEK293 cells. Co‐IP assays revealed that the C‐terminal mutation construct containing aa 924–1561, which included the whole segment truncated in GLTSCR1^FS^, bound to BRD4, as did the construct containing aa 1161–1561 and full length of GLTSCR1, but the interaction with BRD4 was not observed for the C‐terminal mutation construct containing aa 1361–1561, although this construct included the NLS (Figure 4D) Thus, we defined aa 1161–1361 as the BRD4 binding domain (BB domain) of GLTSCR1, which was consistent with previous study that defined 1297–1321 peptide of GLTSCR1 as a recognition region for BRD3.^19^

As an epigenetic reader protein, BRD4 plays an oncogenic role in cancer metastatic processes and clinical stages through binding to acetylated lysine residues on histones.^16^ To explore whether GLTSCR1 inhibits CRC metastasis through interaction with BRD4, we used shRNA to stably knock down BRD4 expression in wild‐type and GLTSCR1‐KO HCT116 cells (Figure 4E). shBRD4 dramatically inhibited cell migration and invasion in both cell types (Figure 4F), suggesting that BRD4 promotes CRC migration and invasion independent of GLTSCR1 binding. However, stably knockdown of GLTSCR1 expression in BRD4‐knockdown HCT116 cells failed to promote cell migration and invasion, indicating that BRD4 plays a critical mediating role in the repression of CRC metastasis by GLTSCR1 (Figure 4G). Taken together, these data clarify that the antimetastatic role of GLTSCR1 in CRC is dependent on its interaction with BRD4 through its C‐terminal BB domain.

### 2.5 The Bromodomains and Phosphorylation‐Dependent Interaction Domain (PDID) of BRD4 are Required for the Interaction with GLTSCR1

**Figure 5:**
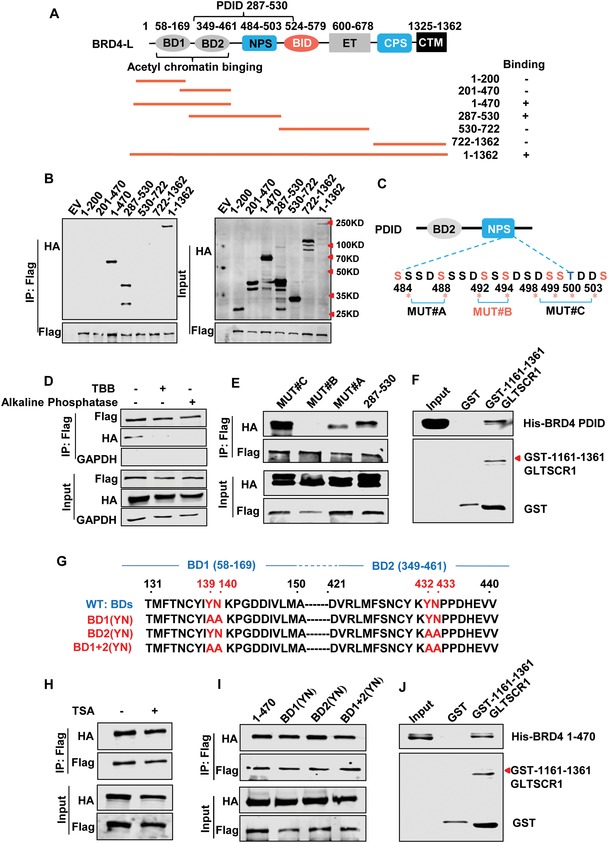
GLTSCR1 binds to the specific domains of BRD4. A) Schematic of the functional domains of BRD4 and the deletion constructs (PDID, phosphorylation‐dependent interaction domain). B) Immunoblotting to detect the immunoprecipitation of exogenous Flag‐tagged GLTSCR1 and HA‐tagged BRD4 with the full‐length sequence or deletion mutations by an anti‐Flag antibody in HEK293 cells. C) Schematic of phosphorylation sites in the PDID and mutation constructs. Seven putative CK2 phosphorylation sites are located at aa 484, 488, 492, 494, 498, 499, and 503. MUT#A (S484A + S488A), MUT#B (S492A + S494A), and MUT#C (S498A + S499A + T500A + S503A). D) Immunoblotting to detect the immunoprecipitation of exogenous Flag‐tagged GLTSCR1 and HA‐tagged BRD4 (PDID) by an anti‐Flag antibody in HEK293 cells treated with TBB (50 × 10^−6^ m) for 6 h and protein lysate treated with alkaline phosphatase for 15 min. E) Immunoblotting to detect the immunoprecipitation of exogenous Flag‐tagged GLTSCR1 and HA‐tagged BRD4 (PDID), MUT#A, MUT#B, or MUT#C by an anti‐Flag antibody in HEK293 cells. F) GST pull‐down assay to detect the interaction between purified GST‐GLTSCR1 (aa 1161–1361) and His‐BRD4 (PDID). G) Schematic of the tyrosine and asparagine residues in the acetyl‐lysine binding pocket in the BDs and the mutation constructs. BD1 (YN) mutation, Y139A/N140A; BD2 (YN) mutation, Y432A/N433A; BD1 + 2 (YN) mutation, Y139A/N140A/Y432A/N433A. H) Immunoblotting to detect the immunoprecipitation of exogenous Flag‐tagged GLTSCR1 and HA‐tagged BRD4 by an anti‐Flag antibody in HEK293 cells treated with TSA (20 × 10^−6^ m) for 12 h. I) Immunoblotting to detect the immunoprecipitation of exogenous Flag‐tagged GLTSCR1 and HA‐tagged BD1 (YN), BD2 (YN), or BD1 + 2 (YN) by an anti‐Flag antibody in HEK293 cells. J) GST pull‐down assay to detect the interaction between purified GST‐GLTSCR1 (aa 1161–1361) and His‐BRD4 (BD1 + BD2).

To further identify the specific GLTSCR1‐binding domains of BRD4, we generated several deletion constructs of BRD4 (**Figure** **5**A and Figure S4A, Supporting Information) considering its domain structure^20^ and then coexpressed these constructs with GLTSCR1 in HEK293 cells. Both BDs (BD1 and BD2, aa 1–470) and the PDID (287–530) retained the ability to interact with GLTSCR1^WT^ (Figure 5B and Figure S4B, Supporting Information). As shown in Figure 5C and Figure S4C in the Supporting Information, the PDID of BRD4, which contains seven putative CK2 phosphorylation sites (pS/TxxE/D), is a highly conserved region in BET family proteins.^21^ To validate whether the interaction between the PDID of BRD4 (PDID‐BRD4) and GLTSCR1 is dependent on the CK2 phosphorylation sites, we used the CK2‐specific inhibitor 4,5,6,7‐tetrabromobenzotriazole (TBB)^22^ to inactivate phosphorylation and alkaline phosphatase to dephosphorylate these phosphorylation sites. The co‐IP results revealed that PDID‐BRD4 could not bind to GLTSCR1 after both TBB and alkaline phosphatase treatment (Figure 5D). Furthermore, the endogenous interaction between BRD4, GLTSCR1 and pTEFb were also decreased by TBB treatment (Figure S4D, Supporting Information). Intriguingly, only mutation of the CK2 phosphorylation sites at Ser492 and Ser494 (S492A and S494A, respectively) on BRD4 can directly disrupt the interaction with GLTSCR1 (Figure 5E and Figure S4E, Supporting Information). To define the direct interaction of these proteins, we further purified the glutathione S transferase (GST)‐tagged GLTSCR1 BB domain and the His‐tagged truncated PDID‐BRD4 protein. The results of the GST pull‐down assay showed a direct reciprocal interaction between PDID‐BRD4 and the GLTSCR1 BB domain (Figure 5F). Therefore, the Ser492 and Ser494 phosphorylation sites on BRD4 were required for PDID‐BRD4 binding to GLTSCR1.

In addition to the PDID, the BDs could bind to GLTSCR1. As shown in Figure 5G, every BD comprises conserved tyrosine and asparagine residues, which form an acetyl‐lysine binding pocket and are responsible for acetylated histone binding. To investigate whether the interaction between these domains and GLTSCR1 is dependent on acetylation, HEK293 cells coexpressing GLTSCR1 and the BRD4‐BDs were treated with the histone deacetylase inhibitor trichostatin (TSA). The co‐IP results showed that TSA did not disrupt this interaction (Figure 5H). Furthermore, we mutated the tyrosine and asparagine residues in either BD1 or BD2 or in both BD1 and BD2 (Figure 5G). Similar to TSA treatment, these mutations did not affect the interaction between GLTSCR1 and the BRD4‐BDs (Figure 5I). Moreover, we deleted aa 292–299 in the BRD4 BDs (∆292–299, Figure S4F, Supporting Information), which is an evolutionarily conserved region in all BET family members and maintains BRD4 stability,^16^ but this deletion did not abolish the interaction (Figure S4G, Supporting Information). The GST pull‐down assay results further confirmed that the BRD4 BDs can directly bind to the BB domain of GLTSCR1 (Figure 5J). Collectively, these results indicate that binding of the BRD4‐BDs to GLTSCR1 operates through an acetyl‐lysine binding pocket‐independent mode.

Furthermore, we used the iterative threading assembly refinement (I‐TASSER) Suite to predict the protein structures of BRD4 (aa 1–540) and GLTSCR1 (aa 1161–1361) and employed ZDOCK to generate an interactive docking prediction of the BRD4‐GLTSCR1 complex and symmetric multimers. Both BD1 and BD2 in our BRD4 (aa 1–540) model overlapped with the crystal structures in the Protein Data Bank. Specifically, the root mean square deviation (RMSD) of BD1 (5DW2) was 2.07 Å, and the RMSD of BD2 (6C7Q) was 1.60 Å, indicating that the modeling process was reliable (Figure S5A, Supporting Information). In the conformation maintained by aa 1–540 of BRD4, the BD2 region was predicted to combine with aa 1161–1181, aa 1241–1260, and aa 1304–1320 of GLTSCR1. In addition, aa 470–500 (the CK2 phosphorylation sites) of BRD4 bound closely to GLTSCR1, especially the Ser492 and Ser494 sites, which were located in the critical binding area and fit in the groove formed by aa 1215–1220 and aa 1280–1295 of GLTSCR1 (Figure S5B, Supporting Information), thus played a key role in the binding of BRD4 and GLTSCR1.

### 2.6 GLTSCR1 Interacts with BRD4 to Regulate Transcriptional Elongation

**Figure 6:**
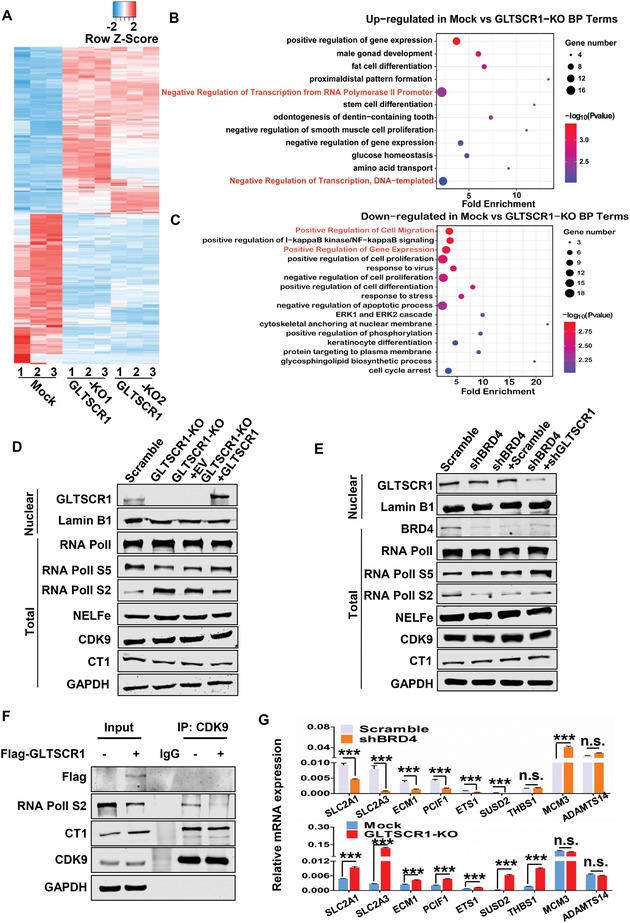
Identification of genes and pathways coregulated by GLTSCR1 and BRD4. A) Heat map of DEGs in HCT116 mock versus GLTSCR1‐KO1 and GLTSCR1‐KO2 cells identified via RNA‐seq. B) Top upregulated biological process terms regulated by GLTSCR1 identified via gene ontology enrichment analysis of the DEGs based on DAVID online tools (*P* < 0.01). C) Top downregulated biological process terms regulated by GLTSCR1 (*P* < 0.01). D) Immunoblotting analysis of transcription elongation complex members in HCT116 cells with GLTSCR1 knockout and re‐expression. E) Immunoblotting analysis of transcription elongation complex members in BRD4 knockdown and GLTSCR1‐BRD4 double knockdown in HCT116 cells. F) Immunoblotting to detect the immunoprecipitation of endogenous CDK9 with CT1, Ser2 phosphorylation site of RNA Pol II and exogenous Flag‐tagged GLTSCR1 by an anti‐CDK9 antibody with or without GLTSCR1 expression in GLTSCR1‐KO HCT116 cells. G) RT‐qPCR to determine the relative mRNA expression of oncogenes coregulated by GLTSCR1 and BRD4, including SLC2A1, SLC2A3, ECM1, PCIF1, ETS1, SUSD2, THBS1, MCM3, and ADAMTS14. Data are presented as the mean ± SD; statistical significance was assessed by an unpaired *t*‐test. *P < 0.05, **P < 0.01, ***P < 0.001; *n* = 3.

As a chromatin adapter, BRD4 can recognize acetylated H3 K14 or H4 K5 or K12 histone marks by its bromodomain^23^ and modulate RNA polymerase II (Pol II)‐mediated transcriptional elongation through positive regulation of pTEFb.^24^ To explore the molecular basis of the interaction between GLTSCR1 and BRD4 in CRC metastasis suppression, we performed genome‐wide transcriptional analysis in two HCT116 GLTSCR1‐KO cell lines by RNA sequencing (RNA‐seq). Two GLTSCR1‐KO cells exhibited 803 overlapped differentially expressed genes (DEGs) (**Figure** **6**A). Via gene ontology (GO) enrichment analysis based on online tools in the Database for Annotation, Visualization, and Integrated Discovery (DAVID), the top upregulated DEGs in both GLTSCR1 knockout cell lines were enriched in several important transcriptional regulation‐associated biological processes (BPs), including Negative Regulation of Transcription from RNA Pol II Promoter, Negative Regulation of Transcription DNA‐templated (Figure 6B). Moreover, the top downregulated DEGs were enriched in the Positive Regulation of Cell Migration and Positive Regulation of Gene Expression, consistent with the function of GLTSCR1 in inhibiting CRC cell migration and invasion (Figure 6C). Interestingly, gene set enrichment analysis (GSEA) showed enrichment in the terms Actin Filament binding, Extracellular Matrix Component, RNA Pol II Activating Transcription Factor Binding, and P53 Pathway (Figure S6A, Supporting Information). Therefore, we presume that GLTSCR1 interaction with BRD4 inhibits CRC metastasis through negatively regulating the transcriptional elongation of oncogenes.

Previous studies showed that RNA Pol II phosphorylation at Ser5 promotes transcription initiation but causes RNA Pol II to pause and accumulate in the proximal promoter region. After the reversal of Ser5 phosphorylation and the induction of pTEFb‐mediated phosphorylation of the RNA Pol II CTD at Ser2 by CDK9, RNA Pol II is released from the initiation site and transcription begins.^25^ To validate whether GLTSCR1 could regulate transcriptional elongation, we assessed RNA Pol II phosphorylation at Ser5 and Ser2 and explored the expression of negative elongation factor (NELFe), CDK9 and CT1 in GLTSCR1 knockout cells and in cells with GLTSCR1 re‐expression. Immunoblotting analysis showed that GLTSCR1 knockout induced RNA Pol II phosphorylation at Ser2 but inhibited Ser5 phosphorylation, re‐expression of GLTSCR1 in GLTSCR1 knockout cells rescued RNA Pol II phosphorylation at Ser5 but inhibited Ser2 phosphorylation. However, the expression levels of NELFe, CDK9, and CT1 remained unchanged (Figure 6D). To determine whether this regulation of GLTSCR1 was dependent on BRD4, we further detected the RNA Pol II phosphorylation at Ser5 and Ser2 in BRD4 knockdown and BRD4‐GLTSCR double knockdown cells. The results showed that the RNA Pol II phosphorylation at Ser2 was decreased and phosphorylation at Ser5 was increased when BRD4 knockdown, but knockdown GLTSCR1 in BRD4 knockdown cells did not change the phosphorylation level at Ser2 and Ser5 (Figure 6E). Furthermore, the endogenous interaction between CDK9 and Ser2 phosphorylation site of RNA Pol II was inhibited by overexpression of GLTSCR1 in GLTSCR1‐KO cells, whereas the re‐expression of GLTSCR1 did not affect interaction between CDK9 and CT1 (Figure 6F and Figure S6B, Supporting Information). Interestingly, GLTSCR1 knockout enhanced Ser5 site specific phosphatase SCP2 to bind to RNA Pol II, which resulted in the dephosphorylating RNA Pol II at Ser5, while the interaction between another Ser5 site specific phosphatase SSU72 and RNA Pol II was not affected (Figure S6C, Supporting Information). Then, we analyzed the DEGs resulting from BRD4 knockdown in HCT116 cells via RNA‐seq (Figure S6D, Supporting Information). These 701 DEGs were enriched in the terms regulation of Cell Migration, Positive Regulation of Gene Expression, Cell Proliferation, DNA Replication, and DNA Damage Response (Figure S6E, Supporting Information), indicating that BRD4 plays multiple roles in carcinogenesis, as previously reported.^16^, ^26^ Since BRD4 positively regulates transcriptional elongation, we investigated the DEGs coregulated by BRD4 and GLTSCR1. We identified 88 overlapping DEGs (Figure S7A,B, Supporting Information). We used ClueGO^27^ and CluePedia^28^ to analyze these 88 coregulated genes to reveal the potential biological roles, and their molecular function (MF) was enriched for Promoter‐specific chromatin binding (Figure S7C, Supporting Information). Among these 88 coregulated genes, 38 genes expression showed a significant negative correlation between GLTSCR1‐KO and shBRD4 cells (Figure S7D, Supporting Information). To validate these DEGs identified by RNA‐seq, the expression of *SLC2A1*, *SLC2A3*, *ECM1*, *PCIF1*, *ETS1*, and *SUSD2* was investigated by RT‐qPCR, at the same time THBS1, MCM3, and ADAMTS14 were also detected as the negative controls. As the major glucose transporters, both SLC2A1 and SLC2A3 enhance glucose uptake in CRC cells, which promotes tumorigenesis.^29^ ECM1 plays a key role in cancer metastatic processes by regulating actin cytoskeletal architecture.^30^ Recent research showed that PCIF1 functioned as a cap‐specific adenosine methyltransferase that was responsible for interacting with Ser5 in the CTD of Pol II and cotranscriptionally introduced N6,2′‐O‐dimethyladenosine (m6Am) modifications in nascent mRNA chains.^31^ ETS1 promotes ovarian cancer metastasis by microenvironmental mechanisms.^32^ In addition, as a Notch3‐regulating gene, SUSD2 promotes ovarian cancer metastasis.^33^ As shown in Figure 6G, these six oncogenes were upregulated when GLTSCR1 was stably knocked out and down regulated when BRD4 was knocked down. However, THBS1 and MCM1 expression was regulated only by GLTSCR1 or BRD4 respectively, ADAMTS14 expression was not changed by both GLTSCR1 and BRD4. Taken together, these results indicate that GLTSCR1 may negatively regulate the transcriptional elongation of target oncogenes to inhibit CRC metastasis through interacting with BRD4 to inhibit CDK9 to phosphorylate RNA Pol II at Ser2 and to compete with SCP2 to maintain the phosphorylation of RNA Pol II at Ser5.

### 2.7 GLTSCR1 Inhibits the Transcriptional Elongation of Target Genes and Enhances CRC Sensitivity to BET Inhibitors

**Figure 7:**
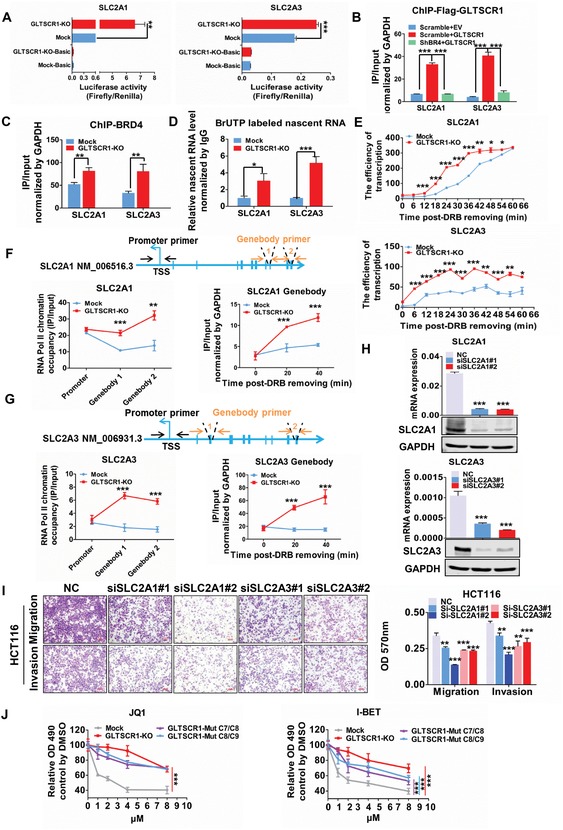
GLTSCR1 regulates transcriptional elongation and enhances CRC cell sensitivity to BET inhibitors A) Luciferase reporter assay to detect the transcriptional activity of SLC2A1 and SLC2A3 in GLTSCR1‐KO HCT116 cells. B) ChIP‐PCR to detect the DNA binding capacity of Flag‐GLTSCR1 to SLC2A1 and SLC2A3 gene in scramble + empty vector, scramble + Flag‐GLTSCR1, and shBRD4 + Flag‐GLTSCR1 cells through pull‐down by anti‐Flag. C) ChIP‐PCR to detect the DNA binding capacity of BRD4 to SLC2A1 and SLC2A3 gene in mock and GLTSCR1‐KO cells through pull‐down by anti‐BRD4. D) Detection for nascent RNA of SLC2A1 and SLC2A3 in mock and GLTSCR1‐KO cells through RNA pull‐down by anti‐BrUTP. E) Transcription efficiency of SLC2A1 and SLC2A3 in GLTSCR1‐KO HCT116 cells, as determined by RT‐qPCR. F) ChIP‐PCR to detect the RNA Pol II chromatin occupancy in multiple sites of SLC2A1 gene in mock and GLTSCR1‐KO cells through pull‐down by anti‐RNA Pol II (left) and to analyze the accumulation of RNA Pol II in gene body of SLC2A1 after releasing from DRB‐inhibition at 0, 20, and 40 min in mock and GLTSCR1‐KO HCT116 cells (right). The upper schematic diagram represents the primers sites of SLC2A1. G) ChIP‐PCR to detect the RNA Pol II chromatin occupancy in multiply sites of SLC2A3 gene in mock and GLTSCR1‐KO cells through pull‐down by anti‐RNA Pol II (left) and to analyze the accumulation of RNA Pol II in gene body of SLC2A3 after releasing from DRB‐inhibition at 0, 20, and 40 min in mock and GLTSCR1‐KO HCT116 cells (right). The upper schematic diagram represents the primers sites of SLC2A3. H) Relative mRNA expression (up) and Immunoblotting analysis (down) of SLC2A1 and SLC2A3 in HCT116 cells transfected with siSLC2A1 and siSLC2A3. I) Transwell assay to investigate the migratory and invasive properties of HCT116 cells transfected with siSLC2A1 and siSLC2A3. The histogram on the right shows the quantification analysis results. J) JQ1 and I‐BET inhibition efficiency in control (mock), GLTSCR1‐KO and GLTSCR1‐C7/C9 heterozygous mutated HCT116 cells. Data are presented as the mean ± SD; statistical significance was assessed by an unpaired *t*‐test. *P < 0.05, **P < 0.01, ***P < 0.001; *n* = 3.

To further investigate the model of GLTSCR1‐mediated regulation of target gene transcriptional elongation, we input the promoter sequences (from positions −2000 to 2000) of these 38 negative correlation coregulated DEGs into the multiple em for motif elicitation (MEME) motif discovery online tool to predict the DNA binding motif of GLTSCR1. Intriguingly, a thymine and adenine (TA)‐enriched motif was discovered between positions −60 to 60 with respect to the transcription start site (TSS) (Figure S7E, Supporting Information), which is exactly the region in which RNA Pol II pauses and accumulates to initiate transcriptional elongation.^25^ Next, we found that both *SLC2A1* and *SLC2A3* contained this specific GLTSCR1 binding motif (Figure S7F, Supporting Information). As previously mentioned, knockout of GLTSCR1 increased the expression of *SLC2A1* and *SLC2A3*. Furthermore, we cloned the *SLC2A1* and *SLC2A3* promoters with the GLTSCR1‐specific motif into a luciferase reporter vector and found that knockout of GLTSCR1 clearly increased the transcriptional activity of *SLC2A1* and *SLC2A3* (**Figure** **7**A). To further verify transcriptional regulation roles of GLTSCR1 through binding to *SLC2A1* and *SLC2A3*, Chromatin immunoprecipitation (ChIP) was performed to demonstrate that GLTSCR1 could bind to the promoter of SLC2A1 and SLC2A3 with the TA‐enriched motif and their affinity were disrupted by BRD4 knockdown (Figure 7B and Figure S8A, Supporting Information). Interestingly, BRD4 shared the same binding site as GLTSCR1 at the promoter of SLC2A1 and SLC2A3, so GLTSCR1 could block BRD4 to bind the specific DNA motif at chromatin level (Figure 7C and Figure S8B, Supporting Information). To examine whether GLTSCR1 regulates gene expression through mediating transcriptional elongation, we used anti‐BrUTP antibody to pull down the nascent RNA labeled with BrUTP, which showed more SLC2A1 and SLC2A3 nascent RNA were synthesized in GLTSCR1 knockout cells (Figure 7D). Then compound 5,6‐dichlorobenzimidazole 1‐β‐d‐ribofuranoside (DRB) was used to pause RNA Pol II in the proximal promoter region and repress transcriptional elongation;^34^ 100 × 10^−6^ m DRB could almost completely block the transcriptional elongation of *SLC2A1* and *SLC2A3* (Figure S8C, Supporting Information). Serial detection of pre‐mRNAs by RT‐qPCR after discontinuing DRB treatment can be used to evaluate the transcription elongation rate. As expected, the transcription elongation rates of *SLC2A1* and *SLC2A3* were significantly enhanced in GLTSCR1‐KO cells after discontinuation of DRB treatment (Figure 7E). Furthermore, ChIP‐PCR assays showed that more RNA Pol II chromatin occupancy was detected in the gene body of SLC2A1 and SLC2A3 when GLTSCR1 was knocked out (Figure 7F,G). We next used ChIP‐PCR assays combining with DRB releasing to examine RNA Pol II releasing and sliding on gene body, the results revealed that RNA Pol II was enriched in the proximal promoter region with the TA binding motif of GLTSCR1, but no difference between mock and GLTSCR1‐KO cell lines (Figure S8D, Supporting Information). It could be interpreted that the high level accumulation of Pol II pauses in the promoter‐proximal region which is a key rate‐limiting step for transcription.^35^ Interestingly, the enrichment of Pol II was higher in gene body of *SLC2A1* and *SLC2A3* in GLTSCR1‐KO than mock cells (Figure 7F,G). These results were consistent with the previous results that GLTSCR1 knockout cells increased the elongation of *SLC2A1* and *SLC2A3* through releasing Pol II from TSS site to gene body. Since GLTSCR1 can inhibit CRC metastasis, the roles of SLC2A1 and SLC2A3 in CRC metastasis need to be identified. Therefore, we used siRNA to knock down the expression of SLC2A1 or SLC2A3 in HCT116 (Figure 7H) and SW480 (Figure S8E,F, Supporting Information) cells and found that migration and invasion were inhibited in both cell lines (Figure 7I and Figure S8G, Supporting Information). Collectively, these results indicate that GLTSCR1 inhibits CRC metastasis through negatively regulating the transcriptional elongation of its target genes, for example, *SLC2A1* and *SLC2A3*.

JQ1 and I‐BET were developed as potent targeted inhibitors of BET family members, including BRD4, for tumor therapy. The homologous drug OTX015, a first‐in‐class small molecule inhibitor (MK‐8628), has been applied in a clinical phase 1 dose‐escalation study in acute myeloid leukemia, lymphoma, and multiple myeloma.^36^ BET inhibitors exert antitumor effects through their high affinity to the BD pockets.^14^ However, the present results demonstrate that GLTSCR1 binds to the BDs independent of the acetyl‐lysine binding pocket but interacts strongly with the PDID in a manner dependent on the phosphorylation of the Ser492 and Ser494 sites on BRD4. Therefore, we hypothesized that GLTSCR1 might enhance the antitumor effect of JQ1 and I‐BET in CRC. As shown in Figure 7J, knockout of GLTSCR1 or GLTSCR1‐C7/C9 heterozygous mutated HCT116 cells significantly decreased the sensitivity to JQ1 and I‐BET; the IC50 of GLTSCR1‐KO cells increased fourfold for JQ1 and nearly threefold for I‐BET (Figure S8H, Supporting Information). Taken together, these results indicate that GLTSCR1 might be a biomarker for BET inhibitor sensitivity and that GLTSCR1 deficiency caused by frameshift mutation results in BET inhibitor resistance in CRC.

## 3 Discussion

**Figure 8:**
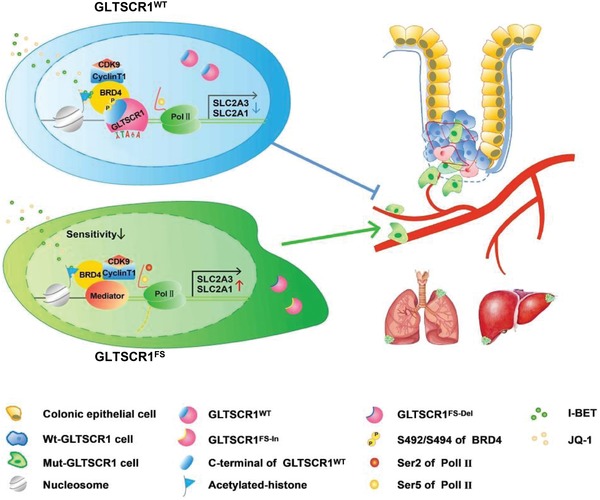
Schematic model of the mechanism of GLTSCR1‐BRD4 complex. In GLTSCR1 wild‐type cells, the BB domain in the C‐terminus of GLTSCR1 binds to the PDID and both BDs in BRD4. Through recognizing the TA‐enriched motif between positions −60 and 60 relative to the TSS of target genes, the GLTSCR1‐BRD4 complex inhibits pTEFb‐mediated phosphorylation of Ser2 in the RNA Pol II CTD and negatively regulates the transcriptional elongation of target oncogenes, such as *SLC2A1* and *SLC2A3*, which play an inhibitory role in CRC metastasis. However, in cells expressing a C8 microsatellite site truncation mutant of GLTSCR1, the frameshift mutation produces two C‐terminal‐truncated GLTSCR1 mutants without the BB domain and NLS. Then, these GLTSCR1 mutants translocate from the nucleus into the cytoplasm. Subsequently, RNA Pol II is phosphorylated at Ser2 by pTEFb, which, in turn, activates SLC2A1 and SLC2A3 transcriptional elongation and promotes CRC metastasis.

CRC is a heterogeneous disease that develops via the interaction of genetic and environmental risk factors. MSI is widely found in CRC as a result of MMR deficiency.^37^ However, the biological consequences and molecular mechanisms of the target genes that are positively selected by MSI during tumor initiation, development, and metastasis remain unclear. In this study, we identified *GLTSCR1*, which is considered as an antimetastatic gene and a novel MSI target gene in CRC. Mechanistically, wild‐type GLTSCR1 binds to BRD4, blocks the pTEFb complex and RNA Pol II and regulates transcriptional elongation. Subsequently, the expression levels of target genes such as *SLC2A1* and *SLC2A3* are reduced, which further inhibits CRC metastasis. The truncated GLTSCR1^FS^ cannot be imported into the nucleus and thus cannot interact with BRD4. Therefore, GLTSCR1^FS^ lose their ability to inhibit of transcription initiation, subsequently promoting CRC metastasis (**Figure** **8**).

Currently, CRCs are believed to arise from three pathways, including the adenoma‐carcinoma sequence (suppressor pathway), the serrated pathway and inherited syndromes such as Lynch syndrome, familial adenomatous polyposis, the human mutY homologue‐associated polyposis, and certain hamartomatous polyposis conditions.^38^ Regardless of the origin of CRC, chromosomal instability, the CpG island methylator phenotype, and MSI have been demonstrated as the three major molecular events in CRC.^39^ Moreover, an MSI‐H phenotype is frequently observed in both sporadic and hereditary CRCs.^5^ Interestingly, frameshift mutations in *GLTSCR1*, which could be considered as a molecular marker of the MSI‐H phenotype, are found only in MSI‐H CRC. These truncated mutations cause a loss of function of GLTSCR1 and promote CRC metastasis, resulting in a poor prognostic outcome. Although the MSI‐H phenotype usually indicates good prognosis in early stage CRC,^40^ patients with MSI‐H metastatic CRC do not appear to have improved outcomes.^41^ In the present study, we found that novel MSI‐H‐specific mutations might contribute to the adverse impact on the outcomes of MSI‐H CRC patients through reversing the antimetastatic roles of GLTSCR1. Although the microsatellite C8 site of GLTSCR1 is a heterozygous mutation, the mutated GLTSCR1 still potently promotes CRC cell metastasis. Because our study showed the expression of GLTSCR1 in CRISPR‐mediated GLTSCR1‐C7/C9 heterozygous mutated HCT116 cells was significantly decreased, this might lead to loss of its function. Another possible reason is the genetic effect of heterozygous GLTSCR1 mutants with a “second hit” from the genetic vulnerabilities created by MSI and MMR deficiency. Therefore, *GLTSCR1* mutation or deletion could be considered as a stratification biomarker for poor prognosis in MSI‐H CRC and should be further investigated.

Although BRD4 lacks a specific DNA binding motif, it plays a key role in transcriptional regulation through interacting with DNA binding proteins.^23^ Encouragingly, we verified the interaction between GLTSCR1 and BRD4 and generated a model for their binding. In this study, we found that the PDID and BDs of BRD4 were two independent regions for binding to GLTSCR1. In the PDID of BRD4, the CK2‐phosphorylated Ser492/Ser494 residues were specific sites for the interaction with GLTSCR1, which could be abolished by treatment with the CK2‐specific inhibitor TBB. Intriguingly, the Ser492 and Ser494 residues are also pivotal sites for the interaction of P53 with BRD4 and the recruitment of p53 for gene promoter regulation.^21^ Consistent with these findings, our RNA‐seq data showed that GLTSCR1‐regulated DEGs were enriched in the p53 pathway. Both *GLTSCR1* and *P53* play tumor‐suppressive roles and share common binding sites in BRD4, which might regulate CRC progression through competitive binding with BRD4. In addition to the PDID, the BDs are other GLTSCR1‐binding core regions in BRD4 that interact with BRD4 independent of the acetyl‐lysine binding pocket. Acetyl‐lysine‐mimicking molecular compounds such as JQ1 and I‐BET can inhibit oncogenic transcriptional programs through synergistically competing with BRD4 in acetyl‐lysine chromatin‐binding modules.^42^ Herein, we reported that GLTSCR1‐KO and CRISPR‐mediated heterozygous mutated CRC cells exhibited decreased sensitivity to JQ1 and I‐BET, indicating that mutations in *GLTSCR1* might be considered as biomarkers for traditional BET inhibitor resistance in CRC. Recently, a class of small peptides that target the PDID phospho‐region on BET was developed.^43^ Although these peptoids are not yet developed for clinical application because of their low affinity for target proteins, they offer a unique strategy to develop approaches for BET inhibition of genes binding specifically to the BRD4 PDID, which will benefit patients with either BRD4‐hyperphosphorylated triple‐negative breast cancer^44^ or GLTSCR1‐mutated CRC as adjuvant chemotherapy in the future.

The pausing of RNA Pol II in proximal promoter regions and its release to initiate a phase of productive elongation are two key rate‐limiting steps in transcriptional regulation.^25^ The pTEFb, as the key regulator of early elongation steps complex, phosphorylates the CTD of RNA Pol II at Ser2, NELFe, and DRB‐sensitivity‐inducing factor; subsequently, RNA Pol II is released from the paused status. BRD4 activates the pTEFb complex by competing with the pTEFb inhibitory complex HEXIM‐7SK.^20^, ^24^ However, we observed that GLTSCR1 directly bind to BRD4 and accumulated in the promoter of target genes through recognition of the specific DNA motif and precise anchoring in the proximal promoter region. Through impeding the interaction of CDK9 and Ser2 site phosphorylated RNA Pol II together with competitive inhibition of SCP2 to bind to RNA Pol II at Ser5, GLTSCR1 reduced phosphorylation of RNA Pol II at Ser2 and increased phosphorylation of RNA Pol II at Ser5, then inhibited target oncogenic transcriptional elongation programs. This biological function of GLTSCR1 is dependent on its interaction with BRD4. As an epigenetics reader, BRD4 recognizes acetylated histone together with transcription factors anchor in DNA. Herein, GLTSCR1 interacted with BRD4 to inhibit its DNA binding effect at chromatin level. However, truncation‐mutated GLTSCR1 loses the potential for BRD4 binding, releases RNA Pol II from the promoter‐proximal pause site to the gene body, following with an active regulation of oncogenic transcriptional elongation, which then promotes the malignant progression of CRC. Moreover, BRD4 plays a versatile role in biological processes, including transcription, chromatin conformation, and DNA damage repair.^26^ Our present study only demonstrated the interaction between GLTSCR1 and BRD4 and the roles of this interaction in regulating gene transcription elongation. However, other biological functions of this interaction are expected to be extensively investigated in the future. To our knowledge, this study is the first to report GLTSCR1 as a CRC suppressor via binding to BRD4 and regulating transcriptional elongation. In addition, the BD domain and PDID domain are conserved in BET family proteins, previous studies characterized BRD9‐GLTSCR1 interaction as the SWI/SNF complex through IP‐mass spectrometry,^45^ and GLTSCR1 was also predicted with a BRD3 recognition region in other cell model.^19^ Therefore, the BRD4‐independent roles of GLTSCR1 in cancer progression and in other diseases need further exploration.

## 4 Conclusion

In summary, we demonstrated that GLTSCR1 negatively regulates oncogenic transcriptional elongation and inhibits CRC metastasis through the binding of its BB domain to both the BDs and the PDID of BRD4. The novel truncated frameshift mutations at the *GLTSCR1* microsatellite site result in the loss of the antimetastatic roles of GLTSCR1 in CRC, which might decrease the sensitivity of CRC cells to BET inhibitors. Our findings highlight the molecular mechanism by which the GLTSCR1‐BRD4 complex coregulates transcriptional elongation, which offers a novel actionable therapeutic target for CRC. Precision therapies with JQ1/I‐BET in GLTSCR1^FS^ CRC patients may reduce off‐target effects and significantly enhance the effectiveness of cancer therapeutics.

## 5 Experimental Section

*Patient Samples*: Colorectal carcinoma samples (tissue resection specimens, *n* = 657) from patients undergoing surgery in the Department of Medicine, Run Run Shaw Hospital, Zhejiang University were included in this study. Research was approved by the Ethics Committee of department of Medicine, Zhejiang University (2018‐018), and all participating patients were informed. Tumor and paired normal tissues and formalin‐fixed paraffin‐embedded samples were prospectively collected from 2007 to 2008.

*Cell Culture and Treatment*: Human colorectal cancer cell lines SW620, SW480, Colo205, DLD‐1, HT29, HCT116, HCT8, HCT15, and RKO were cultured in Roswell Park Memorial Institute (RPMI) 1640 medium, LOVO was cultured in F12 medium. Human HEK293 cell line was cultured in Dulbecco's modified Eagle medium. All media were supplemented with glutamine, 10% fetal bovine serum, and penicillin/streptomycin. Cell lines were grown in a humidified atmosphere at 37C with 5% CO2. SW620, SW480, Colo205, DLD‐1, HT29, HCT116, HCT8, HCT15, and RKO were purchased from the American Type Culture Collection (Manassas, VA, USA). HEK293 was purchased from the cell bank at the Chinese Academy of Sciences (Shanghai, China). Plasmids were transfected into CRC cell lines with Lipofectamine 2000 (Invitrogen), and into HEK293 cells with LipoD293 (SignaGen). SiRNAs were transfected with GenMute siRNA Transfection Reagent (SignaGen, CAT No. SL100568).

*Capillary Electrophoresis and MSI States Analysis*: BAT25, BAT26, D5S346, D17S250, and D2S123 five NCI recommended MSI markers were analyzed by Multi‐PCR of carcinoma and adjacent normal DNA from 657 sporadic CRC samples. Multi‐PCR was performed by using GoTaq Colorless Master Mix (Promega, M7133). Then PCR products were analyzed by capillary electrophoresis (ABI 3130 Genetic Analyzer). Microsatellite instability status was analyzed by Genemapper Analysis software. Samples were classified as MSI‐H with at least two of the five instability markers, MSI‐L with only one marker, and MSS with absence of instability.

*Transwell Migration and Invasion Assay*: Cell motility and invasiveness were measured by transwell and matrigel chamber plates respectively (24‐well format; 8 µm pore size; Corning Costar, New York, USA). 1 × 10^5^ cells were loaded per transwell cultured with serum‐free media in upside of membrane and 10% fetal bovine serum was added at the bottom of the insert. After 18 h for HCT116 or 48 h for SW480 and RKO, the migrated cells were fixed in 4% methanal for 10 min and stained with crystal violet for another 1 h. 30% glacial acetic acid was used to elute crystal violet to quantify the cells on the lower face of the filter. Three independent experiments were performed in triplicate.

*Histological Analysis*: Mice lung, liver, and spleen samples were fixed with 10% neutral buffered formalin and paraffin embedded. 4 µm thick sections were stained with hematoxylin and eosin (H&E) and processed for immunohistochemistry.

After dewaxing, endogenous peroxidase activity was blocked by 3% hydrogen peroxide at room temperature for 15 min. Tissues were treated with citrate buffer (pH 6.0) under high pressure for 3 min to finish antigen retrieval. 10% normal goat serum was used to block for 30 min. Then the sections were incubated with primary antibody overnight at 4 °C (antibody details are provided in the Key Resources Table), followed by incubation with secondary antibodies. Sections were developed with 3,3‐diaminobenzidine (Zhongshan Golden bridge Biotechnology, Beijing, China). Then, sections were counterstained with hematoxylin, dehydrated, and then covered with coverslip.

*Immunoblotting and co‐IP*: Whole cell lysates were extracted with radio‐immunoprecipitation assay buffer and complete protease inhibitor mix (Fdbio science, CAT No. FD009) followed by sonication. Protein concentrations were measured by bicinchoninic acid protein assay (Thermo Scientific, CAT No. 23225). Equal amounts of protein lysates were separated by sodium dodecyl sulfate‐polyacrylamide gel electrophoresis (SDS‐PAGE). After transferring onto nitrocellulose membranes (Millipore), blocking with 5% milk and incubating with antibody overnight at 4 °C, followed by incubation with fluorescence secondary antibodies, detecting by odyssey (LI‐COR).

For co‐IP, cells were lysed in lysis buffer (PH = 8.0, 20 × 10^−3^ m Tris‐HCl, 150 × 10^−3^ m NaCl2, 1.5 × 10^−3^ m ethylenediaminetetraacetic acid (EDTA), 5 × 10^−3^ m ethylenebis(oxyethylenenitrilo)tetraacetic acid (EGTA), 0.5 × 10^−3^ m NaVO4, 0.5% NP‐40 and complete protease inhibitor mix, Roche) followed by sonication. After centrifugation samples were rotated incubating with Anti‐FLAG M2 Magnetic Beads (Sigma) overnight at 4 °C and washed beads by wash buffer (PH = 8.0, 20 × 10^−3^ m Tris‐HCl, 50 × 10^−3^ m NaCl2, 1.5 × 10^−3^ m EDTA, 5 × 10^−3^ m EGTA, 0.5 × 10^−3^ m NaVO4 and 0.5% NP‐40) several times, boiling samples in loading buffer with denaturant SDS. Equal amounts of total protein were immunoprecipitated with antibodies. Precipitates were analyzed by immunoblotting. An aliquot of each lysate was used as input control.

For endogenous co‐IP assay, Bio‐RAD SureBeads Protein A Magnetic Beads (Cat No. 161‐4013) were used to incubate with 2 µg antibody of BRD4, CT1, or CDK9 for 30 min at room temperature, which were washed with PBST (1× phosphate buffer saline (PBS) + 0.1% Tween 20), and mixed with 500 µL cell lysis. After incubating at 4 °C overnight, the mixture was washed by PBST three times and denatured by boiling with denaturant SDS.

*Mice Models for Metastasis*: All animal experiments were performed in accordance with a protocol approved by the Institutional Animal Care and Use Committee at the Zhejiang University. HCT116 mock and HCT116 GLTSCR1‐KO cell lines were infected with lentiviruses of pGKV5 to obtain the cell line stably expressing luciferase. Two groups of cells were injected into immunodeficient mice (NOD–SCID–gamma, male, 4 weeks old) by tail vein. Each mouse was injected with 1 × 10^6^ cells suspend with 100 µL PBS. Mice were intraperitoneal injection with d‐luciferin (300 mg kg^−1^, 5 min prior to imaging), anesthetized with 3% isoflurane, and then imaged in an interactive video information system spectrum imaging system (Caliper, Newton, USA) every week. Images were analyzed with Living Image software (Caliper, Newton, USA). Bioluminescent flux (photons s^−1^ sr^−1^ cm^−2^) was determined for the tumors and lungs. The mice were killed after 40 d and the lungs were removed for histological analysis.

HCT116 mock and HCT116 GLTSCR1‐KO cell lines with stably luciferase expressed were injected into spleen of BALB/c nu/nu nude mice (male, 5 weeks of age) by performing surgery. The mice were killed after 60 d. Before killed, mice were intraperitoneal injection with d‐luciferin (300 mg kg^−1^, 5 min prior to imaging). After killed, liver was separated and detected metastatic foci by bioluminescence imaging. Images were analyzed with Living Image software. Liver and spleen were removed for histological analysis.

*RT‐qPCR and Transcription Elongation Assay*: Total RNA from cells was isolated using the Trizol reagent (Invitrogen/Thermo Fisher Scientific). After reverse transcription, real‐time PCR analysis was performed using synergy brands (SYBR) Premix Ex Taq (RR420A, TaKaRa).

HCT116 mock and HCT116 GLTSCR1‐KO cell lines were seeded in 12 well cell culture plates. After 18 h, all of them were treated with 100 × 10^−6^ m DRB (Sigma, CAT No. D1916) for 3 h, and then washed by PBS for three times, then 1 mL fresh 1640 medium with 10% fetal bovine serum and penicillin/streptomycin was added into cells after DRB removal. The cells were harvested at 5 min intervals for RNA isolation and quantitative RT‐qPCR.

RT‐qPCR was used to measure the expression levels of SLC2A1 pre‐mRNA in the exon 2 regions of the gene and SLC2A3 pre‐mRNA in the exon 1 regions of the gene. Specially, the primers sites spanning the exon and adjacent intron for pre‐mRNA were designed. The expression values are plotted relative to the expression level of the “no‐treatment” control, which was set to 100% in all experiments.

*Immunofluorescence Assay*: Cells were fixed in 4% paraformaldehyde for 10 min and then permeabilized in 0.1% Triton X‐100 for 10 min. The cells were washed in PBS and then blocked with 10% normal goat serum for 30 min, followed incubated antibody overnight at 4 °C. The slides were then incubated with specific anti‐Mouse Alexa 488 or anti‐Rabbit Alexa 546 secondary fluorescence antibody for 1 h and then incubated with 4,6‐diamino‐2‐phenyl indole (DAPI) (Thermo Fisher) for 20 min. Representative images of the pattern of location of each molecule are shown. All confocal analyses were repeated three times

*Luciferase Reporter Assay*: HCT116 mock and HCT116 GLTSCR1‐KO cell line were cotransfected pGL3‐basic or pGL3‐SLC2A1 and pGL3‐SLC2A3 with pRL‐TK vector which was transfected as an internal reference. Cells were lysed and analyzed using the Dual‐Luciferase Reporter Assay Kit (Promega, E1910) according to the manufacturer's instructions 48 h after transfected.

*GST Pull Down Assay*: In vitro transformed pGEX‐GST‐GLTSCR1 (1161–1361), pET‐28a‐His‐BRD4 (1–470), or pET‐28a‐His‐BRD4 (285–530) vectors to BL21 *Escherichia coli* and cultured for 16 h 240 rpm at 37 °C. After added 0.2 × 10^−3^ m isopropyl‐beta‐d‐thiogalactopyranoside, cultured for another 5 h 180 rpm at 20 °C, then centrifugation and lysis bacteria used lysis buffer (0.5% Triton‐X100, 50 × 10^−3^ m NaH2PO4, 300 × 10^−3^ m NaCl, and complete protease inhibitor mix, Roche). The supernatant was then incubated with the glutathione agarose beads with GST‐fusion or His‐fusion with TALON Metal Affinity Resin beads, purified His‐tagged BRD4 (1–470, BDs domain), His‐BRD4 (258–530, PDID domain), and GST‐GLTSCR1 (1161–1361) proteins. For in vitro interaction studies, purified GST‐GLTSCR1 (1161–1361) protein was incubated with His‐tagged BRD4 (1–470, BDs domain) or His‐BRD4 (258–530, PDID domain) at 4 °C overnight on a rocking platform. The beads were collected by centrifugation at 2000 g for 30 s, washed three times in lysis buffer. The interaction of pET‐28a‐His‐BRD4 (1–470) or pET‐28a‐His‐BRD4 (285–530) and pGEX‐GST‐GLTSCR1 (1161–1361) were determined by immunoblotting analysis.

*Protein Structure Modeling*: As its excellent performance in the prediction of protein structures, I‐TASSER Suite^46^ was employed to predict the protein structure of BRD4 and GLTSCR1 according to the target sequence. As an online platform, I‐TASSER server is implemented on the basis of algorithms for protein structure and function predictions, consisting of four general steps which includes threading template identification, iterative structure assembly simulation, model selection and refinement, and structure‐based function annotation. The 3D structure for 1–540 amino acids of BRD4 and 1161–1361 amino acids of GLTSCR1 were constructed by I‐TASSER Suite through the online server.^46^ Then, the binding mechanism of BRD4 and GLTSCR1 were investigated by ZDOCK,^47^ which provides interactive docking prediction of protein–protein complexes and symmetric multimers. The 3D structures of BRD4 and GLTSCR1 created by I‐TASSER were submitted for protein–protein interaction investigation through the ZDOCK online platform.^47^ In each prediction, ten best models were kept for further analysis.

*RNA Seq, GSEA Analysis, and ClueGO Functional Analysis*: RNA of HCT116 mock, GLTSCR1‐KO1, KO2, HCT116 scramble, and shBRD4 cells was extracted, sequenced, and analyzed by RiboBio (Guangzhou, China). Three biological replicates were used for condition. The cDNA libraries were prepared from high quality RNA using an Illumina TruSeq RNA sample prep kit following the manufacturer's instructions (Illumina, San Diego, CA, USA). The individual RNA seq libraries were pooled based on their respective sample‐specific‐6 bp adaptors and sequenced at 150 bp/sequence pair‐read using an Illumina HiSeq 3000 sequencer. Gene differential expression analysis was accomplished by Cuffdiff program in Cufflinks package. Benjamini–Hochberg false discovery rate method was applied to correct for multiple hypothesis testing. The genes with *P* < 0.05, fold change > 1.5 or fold change < 0.67 were defined as different expression genes as candidates for further analysis. Gene expression Heatmap was drawn with Heatmapper.^48^ Gene Ontology enrichment analysis was performed using DAVID.^49^ The results were visualized by the R package ggplot2 in R software.

GSEA^50^ program was used to analyze gene expression data at the level of gene sets. GSEA was applied to the C5 (GO gene sets) and C6 (oncogenic signatures) gene sets, the gene sets collection database MSigDBv6.2 was used and sets with size of 15 to 500 were selected. The permutation of gene was used to generate null distribution and keep all other parameters in their default.

The biological role of the GLTSCR1 and BRD4 coregulated 88 genes were investigated in ClueGO. Enriched pathways within a network which was interconnected based on the κ score were presented by ClueGO. The size of the nodes shows the term significance after Benjamini–Hochberg correction. GO sets, MF, cellular component, BP, and Kyoto encyclopedia of genes and genomes pathways were used for the analysis. In each set, at least two genes from the initial list representing a minimum of 4% were selected. The node positions were determined by Organic algorithm based on their connectivity was used for organizing the networks.

*MEME Motif Discovery*: Statistically significant downregulated genes were collected from shBRD4 and statistically significant upregulated genes from GLTSCR1‐KO (overlapped by GLTSCR1‐KO1 and GLTSCR1‐KO2) by RNA‐Seq. The promoters from −2000 to 2000 of these 38 genes were input to the de novo motif discovery using MEME tool. This is a variation of the MEME suite (http://meme.nbcr.net/meme/), which allows position‐specific priors to assign a probability that a motif starts at each possible location in a sequence. The training set was used as “positive” and the control as “negative.” The motif size was set to 4–20, to accommodate the expected binding site of a typical DNA‐binding protein.

*Cell Proliferation Assays and Cell Viability Assays*: The indicated cell lines were seeded in 96‐well plates (2 × 10^3^ cells per well) and cultured in 100 µL of complete medium. For cell proliferative ability, 0, 24, 48, 72, and 96 h were set for continuous detection used CCK8 kit (Bioshide) according to the manufacturer's instructions. HCT116 mock, HCT116 GLTSCR1‐KO, and HCT116 C9/C7 heterozygous mutation cell lines were seeded into 96‐well plates and incubated with 100 µL culture medium added with different concentration of JQ‐1 or I‐BET for 48 h then performed CCK8 cell viability assays.

*Colony Formation*: For the short‐term colony formation assay, mock and GLTSCR1‐KO1/KO2 HCT116 cells were seeded in six‐well plates (3000 cells per well) in RPMI 1640 medium and were cultured for 2 weeks. Then cells were fixed by methanol and stained with crystal violet in order to count cell number. Data are shown as mean ± s.d. from three independent experiments.

*Chromatin Immunoprecipitation Assay*: HCT116 scramble and HCT116 shBRD4 cells were seeded in 10 cm culture dish. After 18 h, cells were transfected with Flag‐GLTSCR1 by Lipo2000. 48 h after transfection, cells were prepared for ChIP assay, used anti‐Flag antibody for chromatin pull down. ChIP assays were performed by SimpleChIP Enzymatic Chromatin IP Kit (Magnetic Beads) (Cell Signaling Technology, CAT No. 9003) according to the manufacturer's instructions. Samples were analyzed by real‐time PCR using SYBR Green Power Master Mix following the manufacturer's protocol or by RT‐PCR with agarose gel electrophoresis.

DRB treated RNA Pol II ChIP‐PCR analysis was performed to detect the accumulation of RNA Pol II in SLC2A1 and SLC2A3. Mock and GLTSCR1‐KO cells were treated with DRB (100 × 10^−6^ m) for 3 h and washed by PBS three times. Then DRB was removed and replaced by 10 mL fresh 1640 complete medium. The cells were harvested for ChIP‐PCR analysis pull down by anti‐RNA Pol II antibody after releasing from DRB‐inhibition at 0, 20, and 40 min. Two sets of primers spanning the proximal promoter region and gene body respectively were designed for detecting the accumulation of RNA Pol II in the promoter and gene body of SLC2A1 and SLC2A3.

*Transcriptional Activity Analysis Through Labeling Nascent RNA with BrUTP*: HCT116 mock and HCT116 GLTSCR1‐KO cells were treated with 4 × 10^−3^ m Bromouridine (Aldrich) for 2 h. Total RNA of 1 × 10^7^ cells for each group was isolated using the Trizol reagent (Invitrogen/Thermo Fisher Scientific). 50 µL Magnetic beads (Dynabeads Protein G, 10004D, Invitrogen) and 10 µg (20 µL) of anti‐BrUTP monoclonal antibodies (BD Biosciences, 555627) and aliquot RNA were used to pull down the BrUTP‐labeled nascent RNA, which was ready for further RT‐qPCR.

*Quantifications and Statistical Analysis*: Statistical specifications of each experiment precision measures (mean and ± standard error of mean) and the statistical tests used are provided in the figures and figure legends. Kaplan–Meier survival analysis was performed using the software IBM SPSS Statistics 20 with the Log‐rank (Mantel–Cox) test.

The following designations for levels of significance were used within this manuscript: *P < 0.05; **P < 0.01; ***P < 0.001; ns, not significant.

Details of primer sequence and product information are shown in the Table S3 in the Supporting Information.

## Conflict of Interest

The authors declare no conflict of interest.

## Supporting information

Supporting Information

## References

[1] R. A. Ganai , E. Johansson , Mol. Cell 2016, 62, 745. 27259205 10.1016/j.molcel.2016.05.003

[2] P. M. Boland , M. B. Yurgelun , C. R. Boland , Ca‐Cancer J. Clin. 2018, 68, 217. 29485237 10.3322/caac.21448 PMC5980692

[3] S. He , Z. Zhao , Y. Yang , D. O'Connell , X. Zhang , S. Oh , B. Ma , J. H. Lee , T. Zhang , B. Varghese , J. Yip , S. Dolatshahi Pirooz , M. Li , Y. Zhang , G. M. Li , S. Ellen Martin , K. Machida , C. Liang , Nat. Commun. 2015, 6, 7839. 26234763 10.1038/ncomms8839 PMC4526116

[4] N. F. C. C. de Miranda , M. van Dinther , B. E. W. M. van den Akker , T. van Wezel , P. ten Dijke , H. Morreau , Gastroenterology 2015, 148, 1427. 25736321 10.1053/j.gastro.2015.02.052

[5] H. Yamamoto , K. Imai , Arch. Toxicol. 2015, 89, 899. 25701956 10.1007/s00204-015-1474-0

[6] R. J. Hause , C. C. Pritchard , J. Shendure , S. J. Salipante , Nat. Med. 2016, 22, 1342. 27694933 10.1038/nm.4191

[7] B. Mlecnik , G. Bindea , H. K. Angell , P. Maby , M. Angelova , D. Tougeron , S. E. Church , L. Lafontaine , M. Fischer , T. Fredriksen , M. Sasso , A. M. Bilocq , A. Kirilovsky , A. C. Obenauf , M. Hamieh , A. Berger , P. Bruneval , J. J. Tuech , J. C. Sabourin , F. Le Pessot , J. Mauillon , A. Rafii , P. Laurent‐Puig , M. R. Speicher , Z. Trajanoski , P. Michel , R. Sesboue , T. Frebourg , F. Pages , V. Valge‐Archer , J. B. Latouche , J. Galon , Immunity 2016, 44, 698. 26982367 10.1016/j.immuni.2016.02.025

[8] J. Grimwood , L. A. Gordon , A. Olsen , A. Terry , J. Schmutz , J. Lamerdin , U. Hellsten , D. Goodstein , O. Couronne , M. Tran‐Gyamfi , A. Aerts , M. Altherr , L. Ashworth , E. Bajorek , S. Black , E. Branscomb , S. Caenepeel , A. Carrano , C. Caoile , Y. M. Chan , M. Christensen , C. A. Cleland , A. Copeland , E. Dalin , P. Dehal , M. Denys , J. C. Detter , J. Escobar , D. Flowers , D. Fotopulos , C. Garcia , A. M. Georgescu , T. Glavina , M. Gomez , E. Gonzales , M. Groza , N. Hammon , T. Hawkins , L. Haydu , I. Ho , W. Huang , S. Israni , J. Jett , K. Kadner , H. Kimball , A. Kobayashi , V. Larionov , S. H. Leem , F. Lopez , Y. Lou , S. Lowry , S. Malfatti , D. Martinez , P. McCready , C. Medina , J. Morgan , K. Nelson , M. Nolan , I. Ovcharenko , S. Pitluck , M. Pollard , A. P. Popkie , P. Predki , G. Quan , L. Ramirez , S. Rash , J. Retterer , A. Rodriguez , S. Rogers , A. Salamov , A. Salazar , X. She , D. Smith , T. Slezak , V. Solovyev , N. Thayer , H. Tice , M. Tsai , A. Ustaszewska , N. Vo , M. Wagner , J. Wheeler , K. Wu , G. Xie , J. Yang , I. Dubchak , T. S. Furey , P. DeJong , M. Dickson , D. Gordon , E. E. Eichler , L. A. Pennacchio , P. Richardson , L. Stubbs , D. S. Rokhsar , R. M. Myers , E. M. Rubin , S. M. Lucas , Nature 2004, 428, 529. 15057824 10.1038/nature02399

[9] J. S. Smith , I. Tachibana , U. Pohl , H. K. Lee , U. Thanarajasingam , B. P. Portier , K. Ueki , S. Ramaswamy , S. J. Billings , H. W. Mohrenweiser , D. N. Louis , R. B. Jenkins , Genomics 2000, 64, 44. 10708517 10.1006/geno.1999.6101

[10] A. Alpsoy , E. C. Dykhuizen , J. Biol. Chem. 2018, 293, 3892. 29374058 10.1074/jbc.RA117.001065 PMC5858003

[11] a) J. Y. Yin , Y. G. Ma , U. Vogel , D. H. Liu , Z. X. Sun , Curr. Med. Sci. 2018, 38, 734; 30128886 10.1007/s11596-018-1938-6

[12] X. Ma , T. Du , D. Zhu , X. Chen , Y. Lai , W. Wu , Q. Wang , C. Lin , Z. Li , L. Liu , H. Huang , Oncol. Lett. 2018, 16, 6749. 30405818 10.3892/ol.2018.9490 PMC6202511

[13] S. Rahman , M. E. Sowa , M. Ottinger , J. A. Smith , Y. Shi , J. W. Harper , P. M. Howley , Mol. Cell. Biol. 2011, 31, 2641. 21555454 10.1128/MCB.01341-10 PMC3133372

[14] J. Shi , C. R. Vakoc , Mol. Cell 2014, 54, 728. 24905006 10.1016/j.molcel.2014.05.016 PMC4236231

[15] J. M. Carethers , B. H. Jung , Gastroenterology 2015, 149, 1177. 26216840 10.1053/j.gastro.2015.06.047 PMC4589489

[16] X. Dai , W. Gan , X. Li , S. Wang , W. Zhang , L. Huang , S. Liu , Q. Zhong , J. Guo , J. Zhang , T. Chen , K. Shimizu , F. Beca , M. Blattner , D. Vasudevan , D. L. Buckley , J. Qi , L. Buser , P. Liu , H. Inuzuka , A. H. Beck , L. Wang , P. J. Wild , L. A. Garraway , M. A. Rubin , C. E. Barbieri , K. K. Wong , S. K. Muthuswamy , J. Huang , Y. Chen , J. E. Bradner , W. Wei , Nat. Med. 2017, 23, 1063. 28805820 10.1038/nm.4378 PMC5625299

[17] M. Sanso , R. S. Levin , J. J. Lipp , V. Y. Wang , A. K. Greifenberg , E. M. Quezada , A. Ali , A. Ghosh , S. Larochelle , T. M. Rana , M. Geyer , L. Tong , K. M. Shokat , R. P. Fisher , Genes Dev. 2016, 30, 117. 26728557 10.1101/gad.269589.115 PMC4701974

[18] M. C. Patel , M. Debrosse , M. Smith , A. Dey , W. Huynh , N. Sarai , T. D. Heightman , T. Tamura , K. Ozato , Mol. Cell. Biol. 2013, 33, 2497. 23589332 10.1128/MCB.01180-12 PMC3700095

[19] D. C. C. Wai , T. N. Szyszka , A. E. Campbell , C. Kwong , L. E. Wilkinson‐White , A. P. G. Silva , J. K. K. Low , A. H. Kwan , R. Gamsjaeger , J. D. Chalmers , W. M. Patrick , B. Lu , C. R. Vakoc , G. A. Blobel , J. P. Mackay , J. Biol. Chem. 2018, 293, 7160. 29567837 10.1074/jbc.RA117.000678 PMC5949996

[20] S. Y. Wu , C. M. Chiang , J. Biol. Chem. 2007, 282, 13141. 17329240 10.1074/jbc.R700001200

[21] S. Y. Wu , A. Y. Lee , H. T. Lai , H. Zhang , C. M. Chiang , Mol. Cell 2013, 49, 843. 23317504 10.1016/j.molcel.2012.12.006 PMC3595396

[22] S. Sarno , H. Reddy , F. Meggio , M. Ruzzene , S. P. Davies , A. Donella‐Deana , D. Shugar , L. A. Pinna , FEBS Lett. 2001, 496, 44. 11343704 10.1016/s0014-5793(01)02404-8

[23] C. M. Chiang , F1000 Biol. Rep. 2009, 1, 98. 20495683 10.3410/B1-98 PMC2873783

[24] R. C. Chen , J. H. N. Yik , Q. J. Lew , S. H. Chao , Biomed. Res. Int. 2014, 2014, 1. 10.1155/2014/232870 PMC3925632 24592384

[25] I. Jonkers , J. T. Lis , Nat. Rev. Mol. Cell Biol. 2015, 16, 167. 25693130 10.1038/nrm3953 PMC4782187

[26] J. Zhang , A. M. Dulak , M. M. Hattersley , B. S. Willis , J. Nikkila , A. Wang , A. Lau , C. Reimer , M. Zinda , S. E. Fawell , G. B. Mills , H. Chen , Oncogene 2018, 37, 3763. 29636547 10.1038/s41388-018-0194-3 PMC6101970

[27] G. Bindea , B. Mlecnik , H. Hackl , P. Charoentong , M. Tosolini , A. Kirilovsky , W. H. Fridman , F. Pages , Z. Trajanoski , J. Galon , Bioinformatics 2009, 25, 1091. 19237447 10.1093/bioinformatics/btp101 PMC2666812

[28] G. Bindea , J. Galon , B. Mlecnik , Bioinformatics 2013, 29, 661. 23325622 10.1093/bioinformatics/btt019 PMC3582273

[29] a) A. Nagarajan , S. K. Dogra , L. Sun , N. Gandotra , T. Ho , G. Cai , G. Cline , P. Kumar , R. A. Cowles , N. Wajapeyee , Mol. Cell 2017, 67, 685; 28803777 10.1016/j.molcel.2017.07.014 PMC5567863

[30] L. Gan , J. Meng , M. Xu , M. Liu , Y. Qi , C. Tan , Y. Wang , P. Zhang , W. Weng , W. Sheng , M. Huang , Z. Wang , Oncogene 2018, 37, 744. 29059156 10.1038/onc.2017.363

[31] S. Akichika , S. Hirano , Y. Shichino , T. Suzuki , H. Nishimasu , R. Ishitani , A. Sugita , Y. Hirose , S. Iwasaki , O. Nureki , T. Suzuki , Science 2019, 363, 6423. 10.1126/science.aav0080 30467178

[32] S. Tomar , J. P. Plotnik , J. Haley , J. Scantland , S. Dasari , Z. Sheikh , R. Emerson , D. Lenz , P. C. Hollenhorst , A. K. Mitra , Cancer Lett. 2018, 414, 190. 29174800 10.1016/j.canlet.2017.11.012

[33] Y. Xu , C. Y. Miao , C. J. Jin , C. P. Qiu , Y. N. Li , X. M. Sun , M. Gao , N. Lu , B. H. Kong , Exp. Cell Res. 2018, 363, 160. 29305171 10.1016/j.yexcr.2017.12.029

[34] J. Singh , R. A. Padgett , Nat. Struct. Mol. Biol. 2009, 16, 1128. 19820712 10.1038/nsmb.1666 PMC2783620

[35] K. Adelman , J. T. Lis , Nat. Rev. Genet. 2012, 13, 720. 22986266 10.1038/nrg3293 PMC3552498

[36] S. Amorim , A. Stathis , M. Gleeson , S. Iyengar , V. Magarotto , X. Leleu , F. Morschhauser , L. Karlin , F. Broussais , K. Rezai , P. Herait , C. Kahatt , F. Lokiec , G. Salles , T. Facon , A. Palumbo , D. Cunningham , E. Zucca , C. Thieblemont , Lancet Haematol. 2016, 3, e196. 27063978 10.1016/S2352-3026(16)00021-1

[37] C. J. Punt , M. Koopman , L. Vermeulen , Nat. Rev. Clin. Oncol. 2017, 14, 235. 27922044 10.1038/nrclinonc.2016.171

[38] a) B. T. Dickinson , J. Kisiel , D. A. Ahlquist , W. M. Grady , Gut 2015, 64, 1485; 25994221 10.1136/gutjnl-2014-308075 PMC4765995

[39] The Cancer Genome Atlas Network , Nature 2012, 487, 330.22810696

[40] W. S. Samowitz , K. Curtin , K. N. Ma , D. Schaffer , L. W. Coleman , M. Leppert , M. L. Slattery , Cancer Epidemiol., Biomarkers Prev. 2001, 10, 917. 11535541

[41] J. Goldstein , B. Tran , J. Ensor , P. Gibbs , H. L. Wong , S. F. Wong , E. Vilar , J. Tie , R. Broaddus , S. Kopetz , J. Desai , M. J. Overman , Ann. Oncol. 2014, 25, 1032. 24585723 10.1093/annonc/mdu100 PMC4072907

[42] A. C. Belkina , G. V. Denis , Nat. Rev. Cancer 2012, 12, 465. 22722403 10.1038/nrc3256 PMC3934568

[43] D. Cai , A. Y. Lee , C. M. Chiang , T. Kodadek , Bioorg. Med. Chem. Lett. 2011, 21, 4960. 21742492 10.1016/j.bmcl.2011.06.011 PMC3591469

[44] S. Shu , C. Y. Lin , H. H. He , R. M. Witwicki , D. P. Tabassum , J. M. Roberts , M. Janiszewska , S. J. Huh , Y. Liang , J. Ryan , E. Doherty , H. Mohammed , H. Guo , D. G. Stover , M. B. Ekram , J. Brown , C. D'Santos , I. E. Krop , D. Dillon , M. McKeown , C. Ott , J. Qi , M. Ni , P. K. Rao , M. Duarte , S. Y. Wu , C. M. Chiang , L. Anders , R. A. Young , E. Winer , A. Letai , W. T. Barry , J. S. Carroll , H. Long , M. Brown , X. S. Liu , C. A. Meyer , J. E. Bradner , K. Polyak , Nature 2016, 529, 413. 26735014 10.1038/nature16508 PMC4854653

[45] J. Gatchalian , S. Malik , J. Ho , D. S. Lee , T. W. R. Kelso , M. N. Shokhirev , J. R. Dixon , D. C. Hargreaves , Nat. Commun. 2018, 9, 5139. 30510198 10.1038/s41467-018-07528-9 PMC6277444

[46] J. Yang , R. Yan , A. Roy , D. Xu , J. Poisson , Y. Zhang , Nat. Methods 2015, 12, 7. 25549265 10.1038/nmeth.3213 PMC4428668

[47] B. G. Pierce , K. Wiehe , H. Hwang , B. H. Kim , T. Vreven , Z. Weng , Bioinformatics 2014, 30, 1771. 24532726 10.1093/bioinformatics/btu097 PMC4058926

[48] S. Babicki , D. Arndt , A. Marcu , Y. J. Liang , J. R. Grant , A. Maciejewski , D. S. Wishart , Nucleic Acids Res. 2016, 44, W147. 27190236 10.1093/nar/gkw419 PMC4987948

[49] a) D. W. Huang , B. T. Sherman , R. A. Lempicki , Nucleic Acids Res. 2009, 37, 1; 19033363 10.1093/nar/gkn923 PMC2615629

[50] a) V. K. Mootha , C. M. Lindgren , K. F. Eriksson , A. Subramanian , S. Sihag , J. Lehar , P. Puigserver , E. Carlsson , M. Ridderstrale , E. Laurila , N. Houstis , M. J. Daly , N. Patterson , J. P. Mesirov , T. R. Golub , P. Tamayo , B. Spiegelman , E. S. Lander , J. N. Hirschhorn , D. Altshuler , L. C. Groop , Nat. Genet. 2003, 34, 267; 12808457 10.1038/ng1180

