# Supplementary material for: GLTSCR1 Negatively Regulates BRD4‐Dependent Transcription Elongation and Inhibits CRC Metastasis
Source: Adv Sci (Weinh). 2019 Oct 16;6(23):1901114. doi: 10.1002/advs.201901114 (PMC6891902; doi:10.1002/advs.201901114)
Supplement: Supplementary file 1 — Supporting Information [file ADVS-6-1901114-s001.pdf]

Copyright WILEY-VCH Verlag GmbH & Co. KGaA, 69469 Weinheim, Germany, 2019.

ADVANCED SCIENCE Open Access

Supporting Information

for *Adv. Sci.*, DOI: 10.1002/advs.201901114

GLTSCR1 Negatively Regulates BRD4-Dependent Transcription Elongation and Inhibits CRC Metastasis

Fengyan Han, Lei Zhang, Chaoyi Chen, Yan Wang, Yi Zhang, Lili Qian, Wenjie Sun, Dan Zhou, Beibei Yang, Honghe Zhang, and Maode Lai**

Supporting Information

GLTSCR1 negatively regulates BRD4-dependent transcription elongation and inhibits CRC metastasis

Fengyan Han[1, #], Lei Zhang[2, #], Chaoyi Chen[1], Yan Wang[1], Yi Zhang[1], Lili Qian[1], Wenjie Sun[1], Dan Zhou[1], Beibei Yang[1], Honghe Zhang[1, *], Maode Lai[1, 2, *]

Affiliations

[1] Department of Pathology, Key Laboratory of Disease Proteomics of Zhejiang Province, Research unit of intelligence classification of tumor pathology and precision therapy Chinese Academy of Medical Sciences (2019RU042), School of Medicine, Zhejiang University, Hangzhou 310058, China.

[2] Department of Pharmacology, China Pharmaceutical University, Nanjing, 210009, China.

[#] These authors contribute equally.

*Correspondence: Prof. Honghe Zhang and Prof. Maode Lai, Department of Pathology, Key laboratory of Disease Proteomics of Zhejiang Province, Research unit of intelligence classification of tumor pathology and precision therapy Chinese Academy of Medical Sciences (2019RU042), School of Medicine, Zhejiang University, Hangzhou 310058, China; honghezhang@zju.edu.cn, lmp@zju.edu.cn.

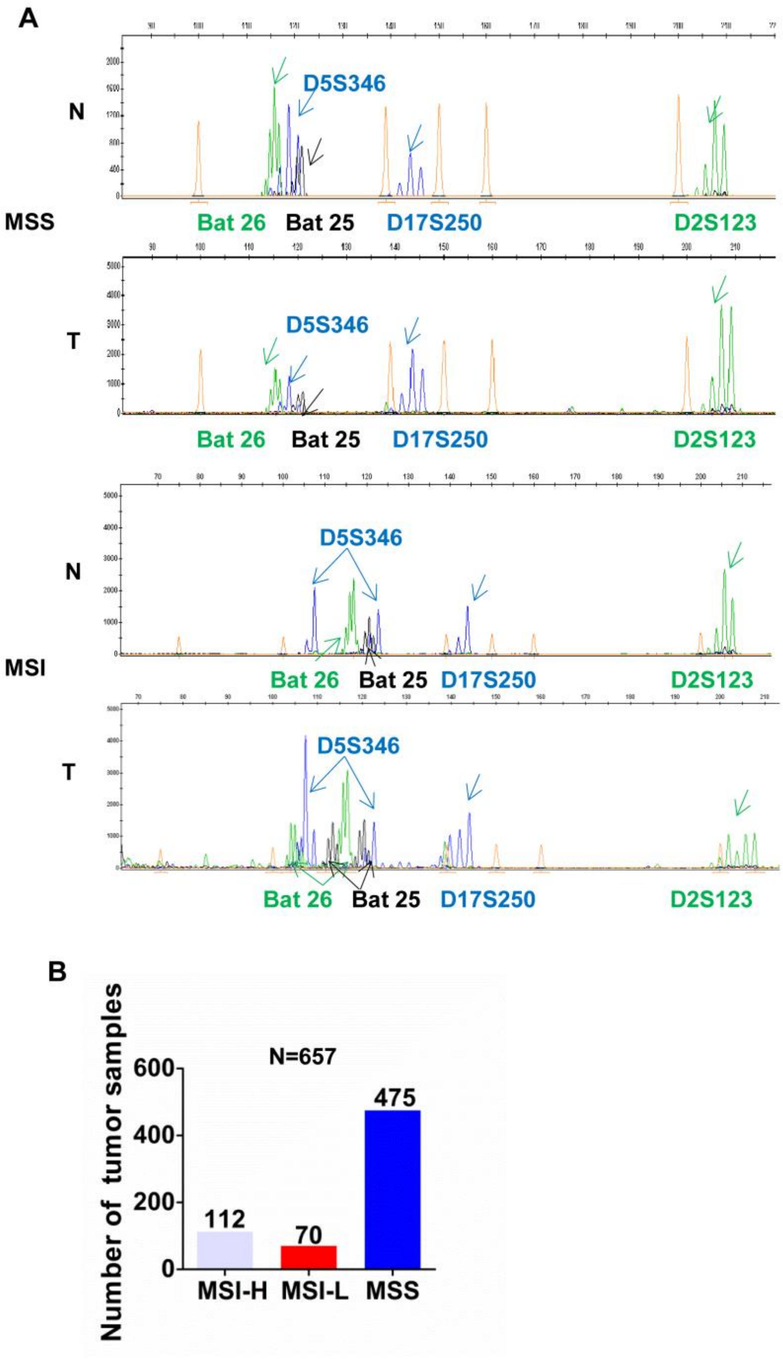

Figure S1: Frameshift mutation in the *GLTSCR1* C8 microsatellite site in CRC

(A) Multiple capillary electrophoresis detection of MSI status using five standard MSI markers. The orange peaks represent the DNA ladder, N, paired normal tissues; T, tumor tissues.

(B) MSI status of CRC samples from our tissue bank.

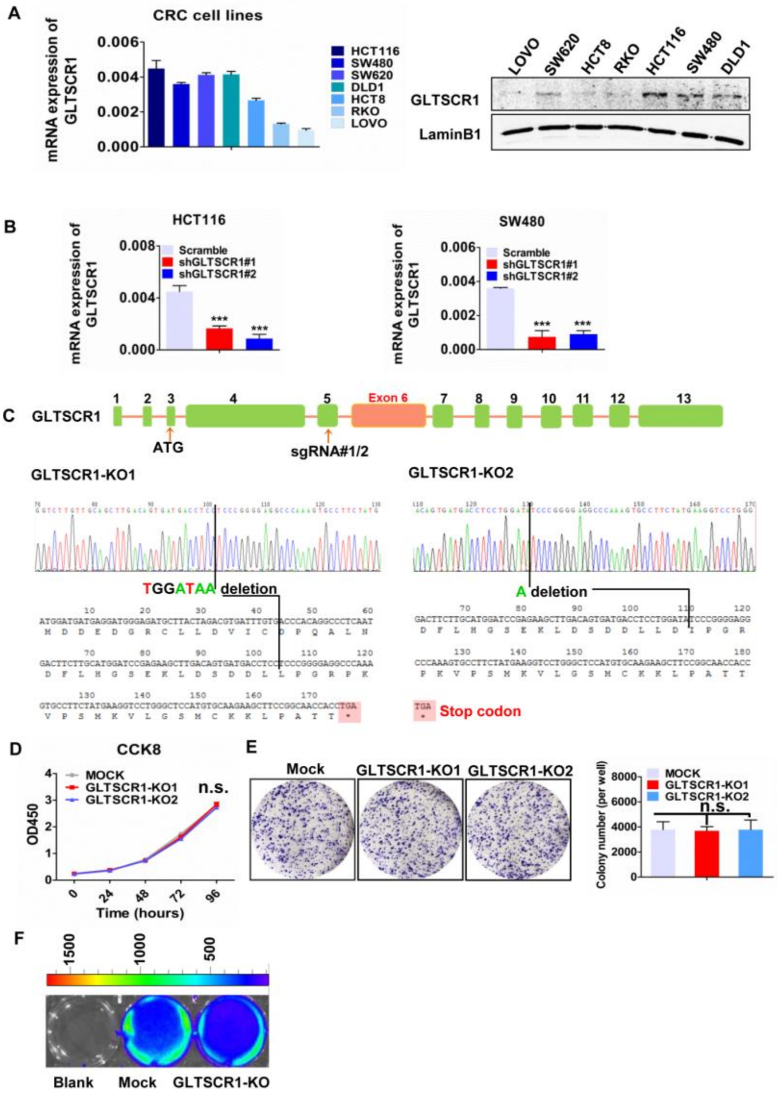

Figure S2: GLTSCR1 inhibits CRC metastasis *in vitro* and *in vivo*

(A) Relative *GLTSCR1* mRNA expression (left) by RT-qPCR and GLTSCR1 protein expression (right) by immunoblotting analysis in CRC cell lines.

(B) Relative mRNA expression of GLTSCR1 in GLTSCR1-knockdown HCT116 and SW480 cells.

(C) Schematic representation of sgRNA-CRISPR/Cas9 target sites in GLTSCR1 and preterminal sites in two stable GLTSCR1-KO cell lines.

(D) Growth curves of control (mock) and GLTSCR1-KO HCT116 cells detected by CCK8 method.

(E) Colony-formation assays of control (mock) and GLTSCR1-KO HCT116 cells, the histograms on the right show the quantification analysis results.

(F) Luciferase signals in control (mock) and GLTSCR1-KO HCT116 cells.

Data are presented as the mean±SD; statistical significance was assessed by an unpaired t-test. *P<0.05, **P<0.01, ***P<0.001; n=3**.**

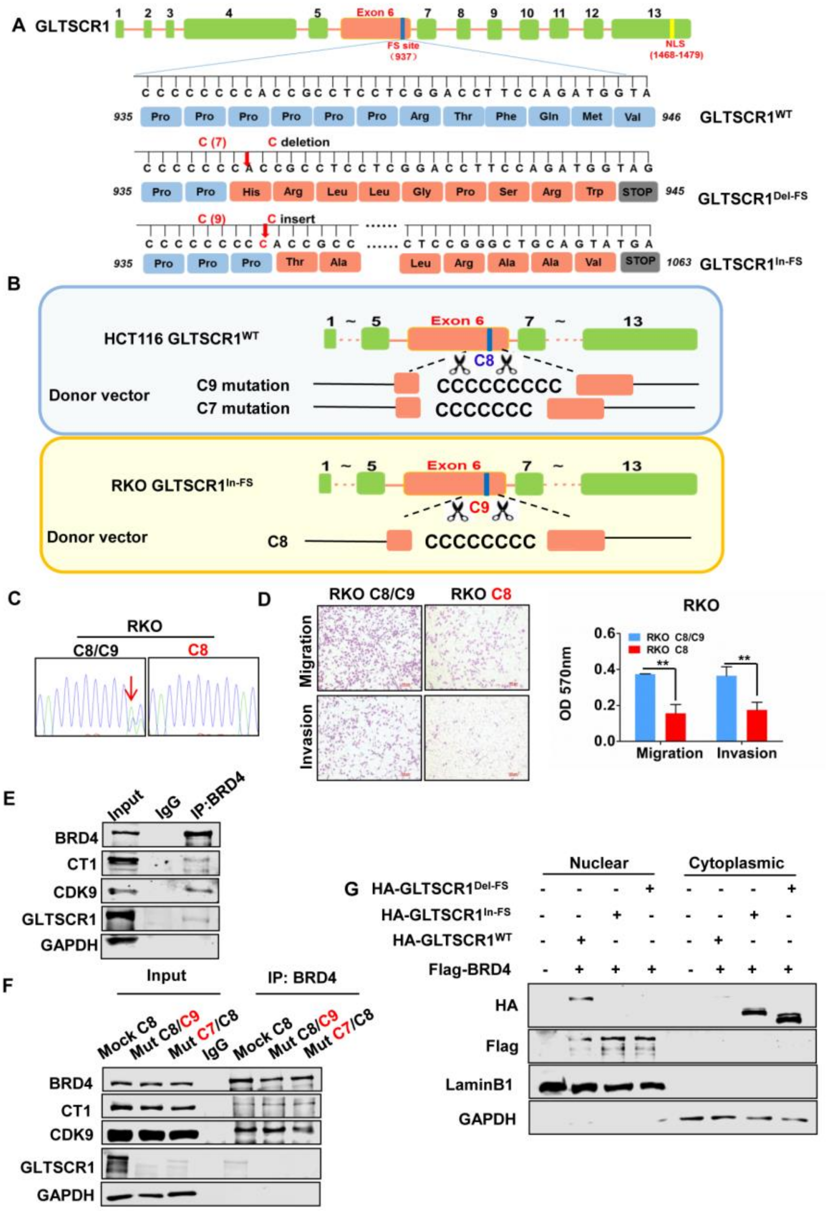

Figure S3: Truncated GLTSCR1^{FS} loses its antimetastatic function.

(A) Schematic diagram of the frameshift mutations in the microsatellite site of *GLTSCR1* exon 6 (generating a premature stop codon (TAG/TGA), thereby producing two types of truncated proteins: GLTSCR1^{Del-FS} and

GLTSCR1^{In-FS}).

(B) Schematic representation of sgRNA-CRISPR/Cas9 target sites in GLTSCR1 and homologous replace fragment sequences in two CRC cell lines.

(C) DNA sequence of the GLTSCR1 C8 microsatellite site in RKO CRISPR-mediated mutated cell lines (the arrows indicate the mutation site).

(D) Transwell assay to investigate the migration and invasion potential of RKO (C8/C9) and CRISPR-rescued mutated RKO cell lines. The histogram on the right shows the quantification analysis results.

(E) Endogenous immunoblotting to detect the immunoprecipitation of BRD4, GLTSCR1, CT1 and CK9 by an anti-BRD4 antibody in HCT116 cells.

(F) Immunoblotting to detect the immunoprecipitation of endogenous GLTSCR1^{FS} and BRD4 in heterozygous MSI-associated GLTSCR1^{FS} mutated HCT116 by an anti-BRD4 antibody.

(G) Immunoblotting to detect subcellular expression of HA-GLTSCR1^{WT}, HA-GLTSCR1^{FS} and Flag-BRD4 in the nuclear and cytoplasm.

Data are presented as the mean±SD; statistical significance was assessed by an unpaired t-test. *$P<0.05$, **$P<0.01$, ***$P<0.001$; n=3**.**

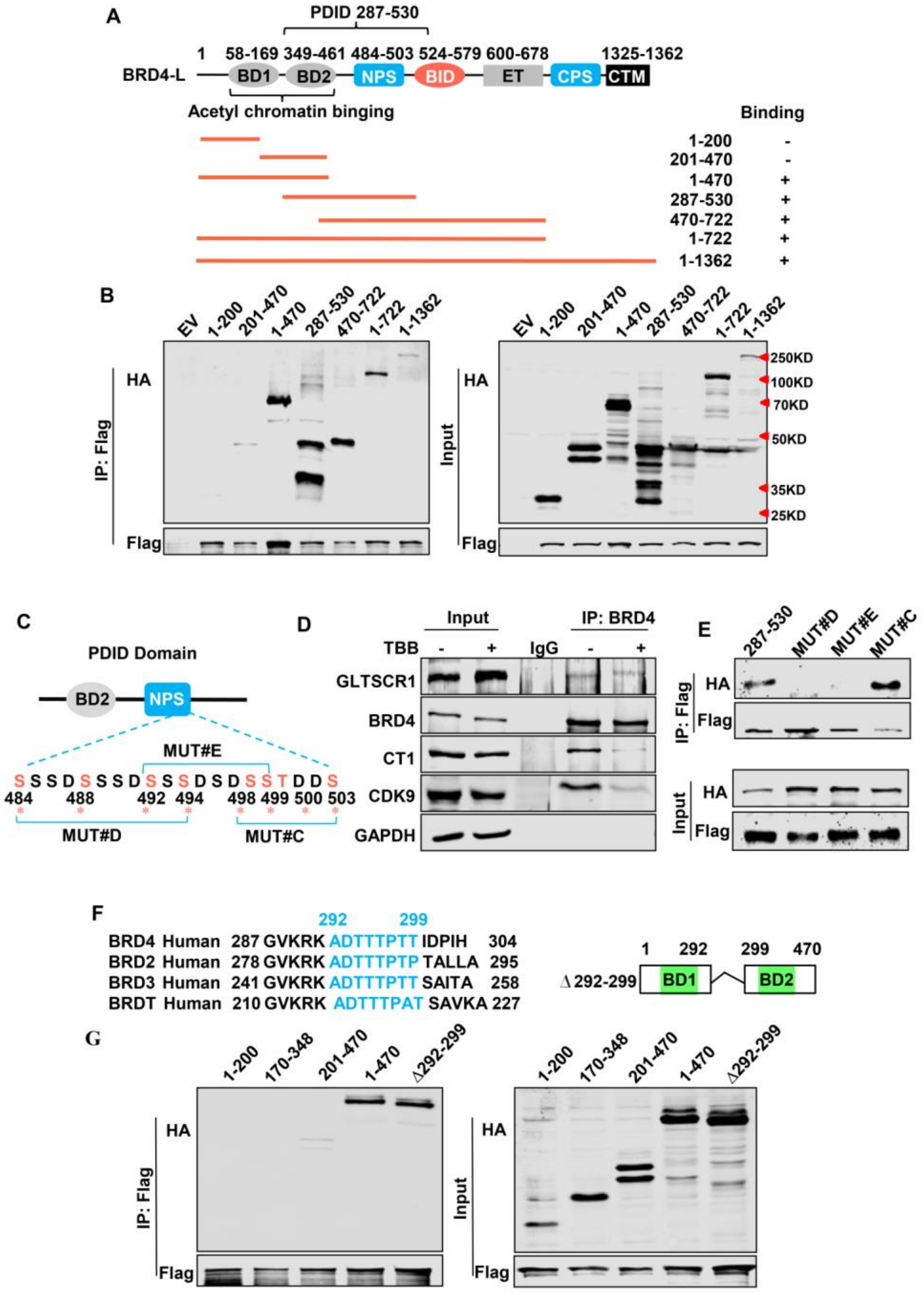

Figure S4: GLTSCR1 binds to the specific domains of BRD4

(A) Schematic of the functional domains of BRD4 and the deletion constructs.

(B) Immunoblotting to detect the immunoprecipitation of exogenous Flag-tagged GLTSCR1 and HA-tagged BRD4 with the full-length sequence or deletion mutations by an anti-Flag antibody in HEK293 cells.

(C) Schematic of the phosphorylation sites in the PDID and the mutant constructs. MUT#D (S484A+S488A+S492A+S494A), MUT#E (S492A+S494A+S498A+S499A), MUT#C

(S498A+S499A+T500A+S503A).

(D) Immunoblotting to detect the immunoprecipitation of endogenous BRD4, GLTSCR1, CT1 and CDK9 by an anti-BRD4 antibody in HCT116 cells treated with TBB (50 µM) for 6 hours.

(E) Immunoblotting to detect the immunoprecipitation of exogenous Flag-GLTSCR1 and HA-tagged BRD4 (PDID), MUT#D, MUT#E or MUT#C by an anti-Flag antibody in HEK293 cells.

(F) Sequence of conserved amino acid sites in BRD4 and a schematic of the deletion mutations.

(G) Immunoblotting to detect the immunoprecipitation of exogenous Flag-GLTSCR1 and HA-tagged BRD4 aa 1-470 or BRD4 deletion mutants by an anti-Flag antibody in HEK293 cells.

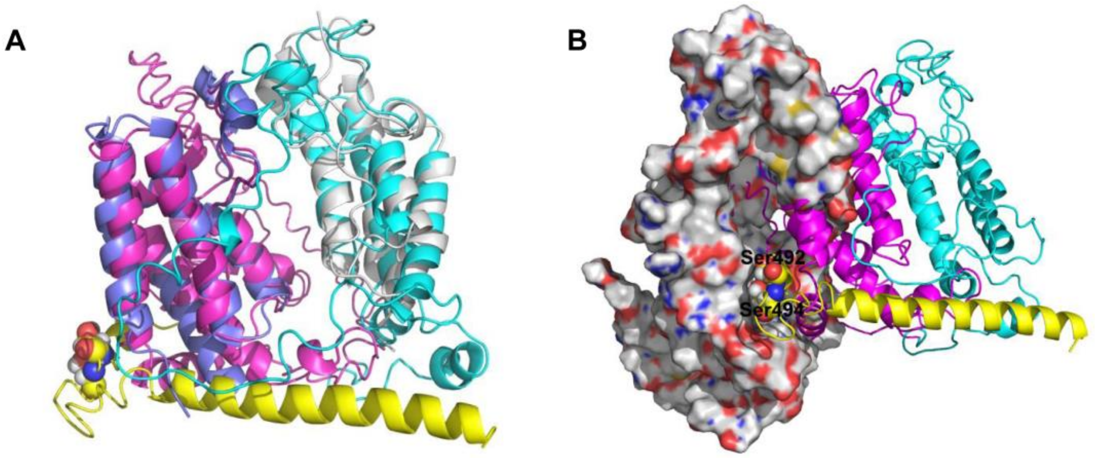

Figure S5: GLTSCR1 binds to the specific domains of BRD4

(A) Alignment of the predicted aa 1-540 of BRD4 with the crystal structures for BRD4 BD1 (aa 42-168; PDB ID 5DW2, gray), and BRD4 BD2 (aa 349-460; PDB ID 6C7Q, dark blue). Among the regions, aa 1-200 of BRD4 are colored aquamarine blue, while aa 201-470 are colored purple. The remaining aa 471-540, are colored yellow. Residues Ser492 and Ser494 are shown as yellow spheres.

(B) Protein-protein docking results for BRD4 and GLTSCR1. The 3D structure of GLTSCR1 (aa 1161-1361) is shown in surface format.

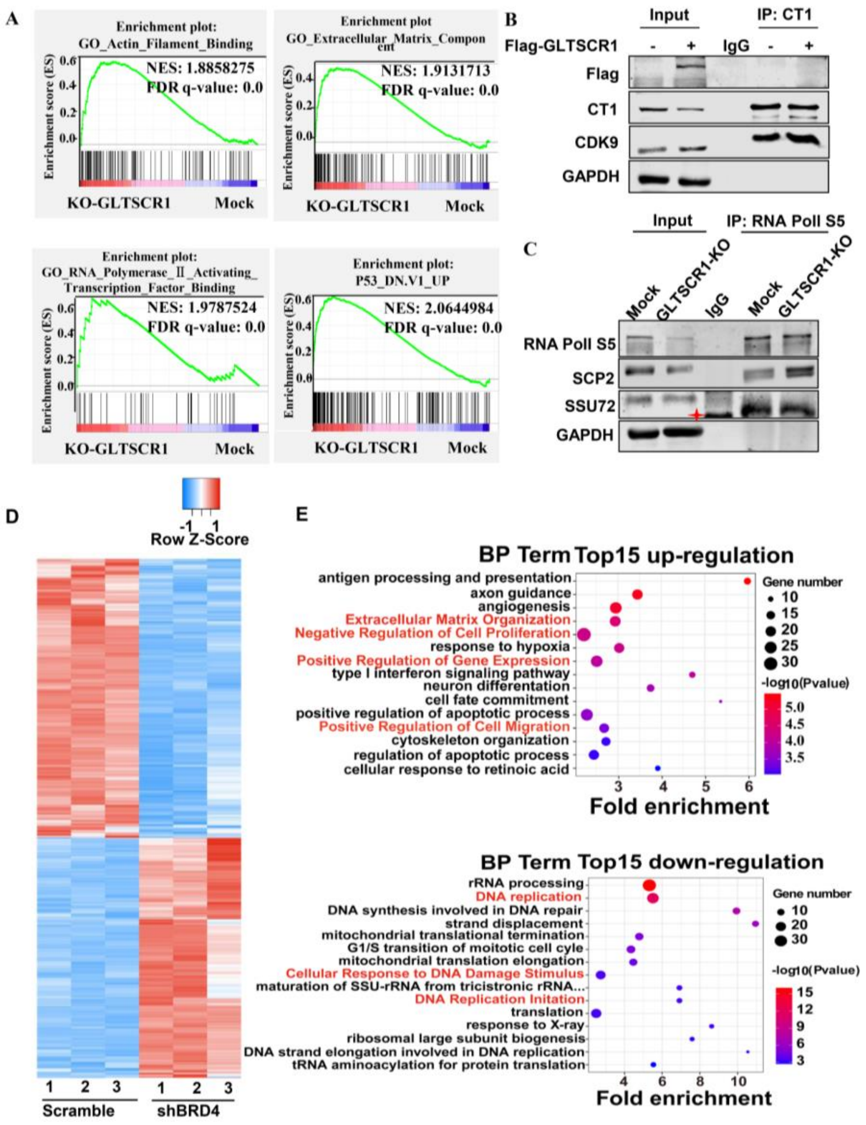

Figure S6: Identification of genes and pathways regulated by GLTSCR1 and BRD4

(A) GSEA of DEGs associated with migration and invasion ability and the P53 pathway.

(B) Immunoblotting to detect the immunoprecipitation of endogenous CT1 with CDK9 and exogenous Flag-GLTSCR1 by an anti-CT1 antibody with or without GLTSCR1 expression in GLTSCR1-KO HCT116 cells.

(C) Immunoblotting to detect the immunoprecipitation of endogenous Ser5 site specific phosphatases SCP2 and SSU72 by an anti-Ser5 of RNA Pol II antibody in mock and GLTSCR1-KO HCT116 cells. The mark indicates the light chain of IgG.

(D) Heat map of DEGs identified in the RNA-seq data between HCT116 cells transfected with scrambled shRNA or shBRD4.

(E) Gene ontology enrichment analysis of the DEGs (C) based on DAVID online tools and Kyoto Encyclopedia of Genes and Genomes (KEGG) pathway analysis. Top upregulated biological process terms and top downregulated biological process terms enriched in DEGs between cells transfected with scrambled shRNA or shBRD4. Several important biological processes are shown in red.

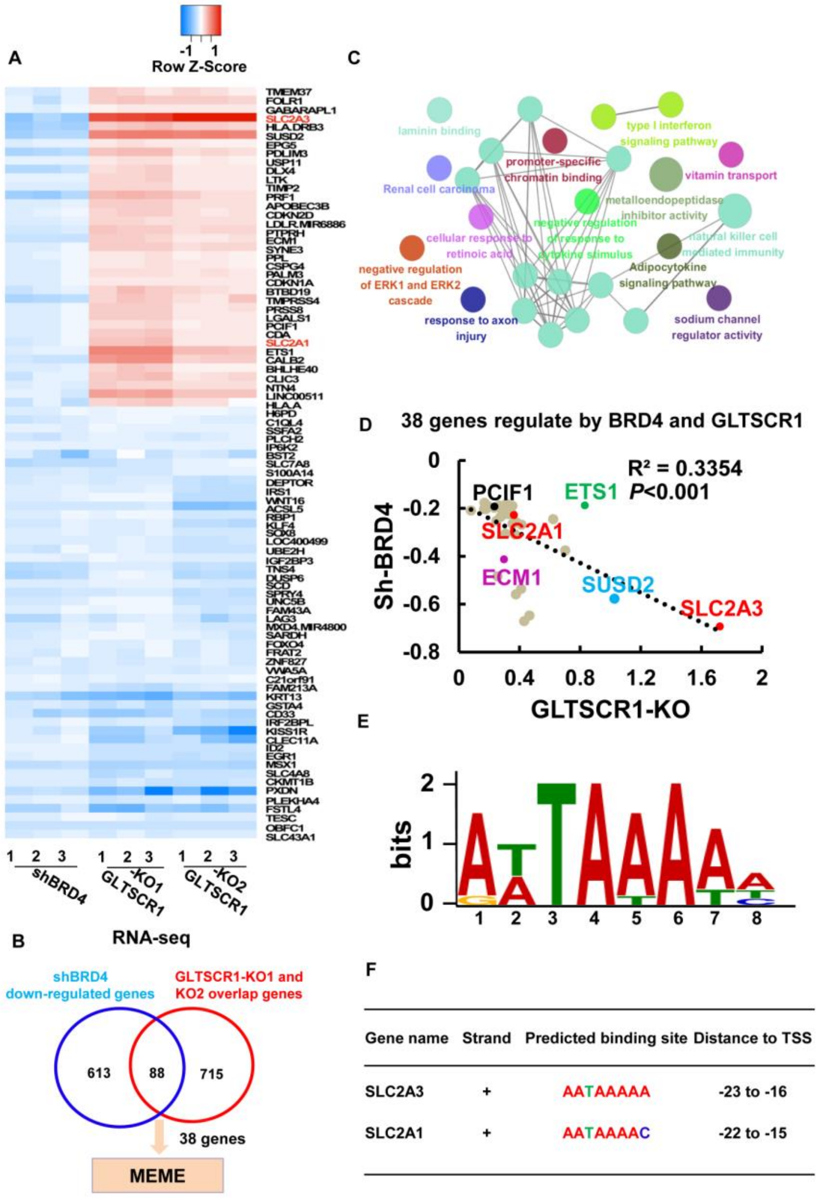

Figure S7: Identification of the target genes and pathways co-regulated by GLTSCR1 and BRD4

(A) Heat map of 88 DEGs co-regulated by BRD4 and GLTSCR1 identified via RNA-seq.

(B) Flowchart of the discovery process for the GLTSCR1 DNA binding motif from the RNA-seq data.

(C) GO Molecular Function and KEGG pathway enrichment of 88 co-regulated genes, which are shown as nodes interconnected based on the κ score. The size of the nodes shows the term significance (Benjamini-Hochberg correction).

(D) The correlation analysis of 38 DEG genes regulate by BRD4 and GLTSCR1. The X and Y axis represent the fold changes in genes regulation.

(E) The TA-enriched motif was discovered by the MEME motif discovery online tool.

(F) Predicted GLTSCR1 binding site in the promoters of SLC2A1 and SLC2A3.

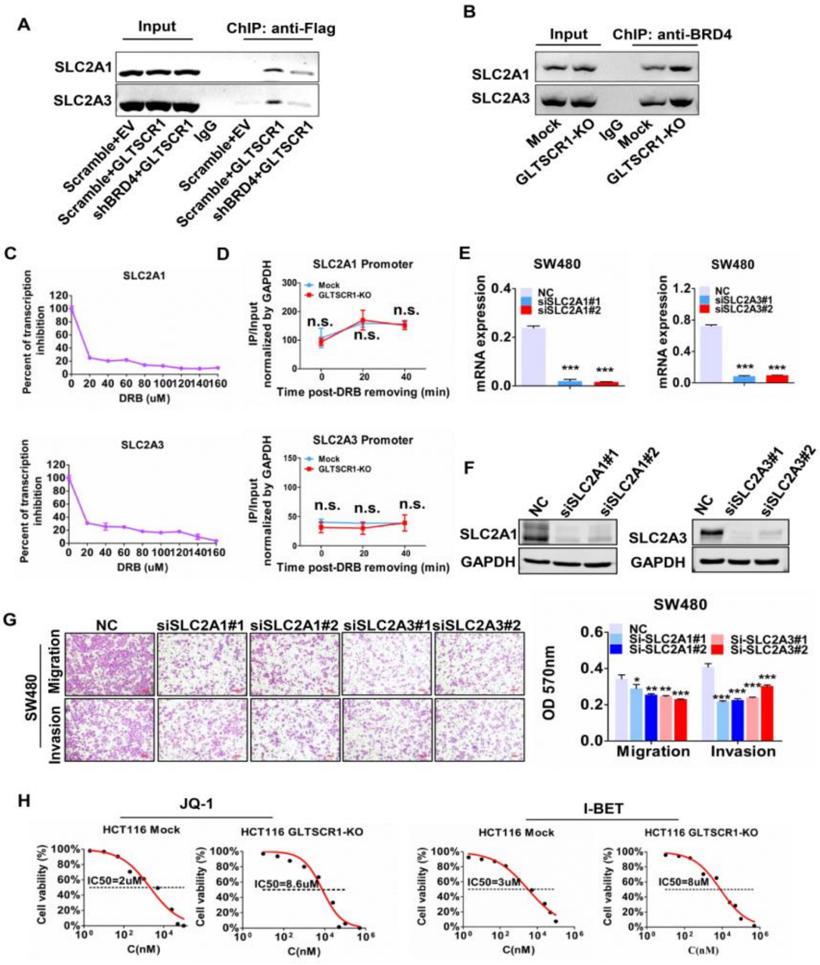

Figure S8: GLTSCR1 regulates transcriptional elongation and enhances CRC cell sensitivity to BET inhibitors

(A) ChIP-PCR to detect the DNA binding capacity of Flag-GLTSCR1 to SLC2A1 and SLC2A3 gene in scramble+EV, scramble+Flag-GLTSCR1 and shBRD4+Flag-GLTSCR1 cells through pull-down by anti-Flag.

(B) ChIP-PCR to detect the DNA binding capacity of BRD4 to SLC2A1 and SLC2A3 gene in mock and GLTSCR1-KO cells through pull-down by anti-BRD4.

(C) Transcription inhibition rates of SLC2A1 and SLC2A3 in HCT116 cells treated with different concentrations of DRB for 3 hours.

(D) ChIP-PCR to detect the accumulation of RNA Pol II in the proximal promoter region of SLC2A1 and SLC2A3 pull down by anti-RNA Pol II antibody after releasing from DRB-inhibition at 0min, 20min and 40min in mock and GLTSCR1-KO HCT116 cells.

(E) RT-qPCR to detect the expression of SLC2A1 and SLC2A3 in control (NC), SLC2A1 knockdown and siSLC2A3 knockdown SW480 cells.

(F) Immunoblotting to detect the expression of SLC2A1 and SLC2A3 in control (NC), SLC2A1 knockdown and siSLC2A3 knockdown SW480 cells.

(G) Transwell assay to investigate the migration and invasion potential of control (NC), siSLC2A1-transfected and siSLC2A3-transfected in SW480 cells. The histogram on the right shows the quantification analysis results.

(G) IC50 of JQ1 and I-BET in control (mock) and GLTSCR1-KO HCT116 cells.

Data are presented as the mean±SD; statistical significance was assessed by an unpaired t-test. *P<0.05, **P<0.01, ***P<0.001; n=3.

Table S1: The frameshift mutation genes in MSI-H group CRC in TCGA and their microsatellite sites

Gene	Location	Start site	End site	Mutation type	Changed base	Samples	Sequence	Role in CRC
RNF43	17	56435161	56435161	Frame_Shift_Del	C	19	ggacccccccgcc	suppressor
TTN	2	179563644	179563644	Splice_Site_Del	A/AAAA	3	actaaaaaaaaaaaaaaa	Unknown
BCL9L	11	118770651	118770652	Frame_Shift_Ins/Del	G	4	ggtgggggggggcag	Oncogene
		118773098	118773098	Frame_Shift_Del	G	4	gctgggggggagg	
SETD1B	12	122242658	122242658	Frame_Shift_Del	C	8	tcaccccccccacc	Unknown
HNRNPL	19	39330959	39330959	Frame_Shift_Del	G	9	ggtgggggggggccc	Unknown
DOCK3	3	51417604	51417604	Frame_Shift_Del	C	12	ataccccccccagc	Unknown
ARID1A	1	27100181	27100182	In_Frame_Ins	GCA	1	gcagcagcagcagcagca	suppressor
MBD6	12	57921002	57921002	Frame_Shift_Del	C	4	acaccccccccagg	Unknown
		57921731	57921732	Frame_Shift_Ins/Del	G	3	cctgggggggggagc	
SRCAP	16	30736370	30736371	Frame_Shift_Ins/Del	C	8	cagccccccccacc	Unknown
SVIL	10	29760115	29760116	Frame_Shift_Ins/Del	C	10	catcccccccctg	suppressor
TEAD2	19	49850472	49850473	Frame_Shift_Ins/Del	G/GG	10	catgggggggggcca	Oncogene
BMPR2	2	203420130	203420130	Frame_Shift_Del	A	7	gggaaaaaaaaccg	suppressor
FLNB	3	58089787	58089788	Frame_Shift_Ins/Del	G	5	catgggggggaca	suppressor
ZC3H18	16	88691140	88691141	Frame_Shift_Ins/Del	C	9	ggaccccccccag	Unknown
GLTSCR1	19	48197890	48197891	Frame_Shift_Ins/Del	C	7	agcacccccccccaccg	suppressor
ADAMTSL4	1	150530506	150530506	Frame_Shift_Del	G	6	tttgggggggggtgg	Unknown
ZNF469	16	88504203	88504203	Frame_Shift_Del	G	1	tcagggggggggcgg	Unknown
BCL9	1	147091500	147091501	Frame_Shift_Ins	C	4	ggaccccccccctccatac	Oncogene
AXIN2	17	63532584	63532585	Frame_Shift_Ins/Del	G	4	ggaccccccccctccatac	suppressor

Table S2: Primers for PCR and siRNA

Gene	Origin	Forward	Reverse	Modify	PCR Type
GLTSCR1	**Human**	**CCCCTACGCCATCCGACT**	**GGGTGGTCACCATCTGGAAG**		**DNA primer**
GLTSCR1	**Human**	**CCCCTACGCCATCCGACT**	**GGGTGGTCACCATCTGGAAG**	**F 5'-FAM**	**DNA primer**
GLTSCR1	**Human**	**ACTGAGTGGCCTGAAGAAGC**	**GCAAGGGTGGTCTTCTCCTC**		**QPCR**
SLC2A1	**Human**	**CTCCCTGCAGTTTGGCTACA**	**GAGACTGTGGGCATGTGTGA**		**Pre-mRNA QPCR**
SLC2A3	**Human**	**GCTGTTTCTGGAATCACCCCT**	**CGTCCTCAGTGGCATATGGTT**		**Pre-mRNA QPCR**
SLC2A1-promoter	**Human**	**TTTGGTTCCTGTGGGTCCTC**	**ACCCCTCTTCATGTCAGGGA**		**ChIP primer**
SLC2A1-genebody	**Human**	**GTATGGGCCCAATGAGCTGT**	**ACCCACAGGAACCAAATCGG**		**ChIP primer**
SLC2A3-promoter	**Human**	**CAAACAGTAGGTTGGCAGTAAGG**	**CAAGTCTCAAAGTCAACAGGCACT**		**ChIP primer**
SLC2A3-genebody	**Human**	**TCTGTTCCTTGCTCTTACAGGATAA**	**AGTTCGGCCACAATAAACCAG**		**ChIP primer**
GAPDH	**Human**	**ACCACAGTCCATGCCATCAC**	**TCCACCACCCTGTTGCTGTA**		**QPCR**
SLC2A1	**Human**	**TTCACTGTCGTGTCGCTGTT**	**GGCCACGATGCTCAGATAGG**		**QPCR**
SLC2A3	**Human**	**GGTGGCTGCTTTATGGGACT**	**GTAAAACCCAGTAGCAGCGG**		**QPCR**
BAT25	**Human**	**TCGCCTCCAAGAATGTAAGT**	**TCTGCATTTTAACTATGGCTC**	**F 5'-NED**	**DNA primer**
BAT26	**Human**	**TGACTACTTTTGACTTCAGCC**	**AACCATTCAACATTTTTAACCC**	**F 5'-VIC**	**DNA primer**
D5S346	**Human**	**ACTCACTCTAGTGATAAATCGGG**	**AGCAGATAAGACAGTATTACTAGTT**	**F 5'-FAM**	**DNA primer**
D17S250	**Human**	**GGAAGAATCAAATAGACAAT**	**GCTGGCCATATATATATTTAAACC**	**F 5'-FAM**	**DNA primer**
D2S123	**Human**	**AAACAGGATGCCTGCCTTTA**	**GGACTTTCCACCTATGGGAC**	**F 5'-VIC**	**DNA primer**
**siRNA**					
**SLC2A1#1**	**Human**	**AAAGAGUGUGCUAAAGAATT**	**UUCUUUAGCACACUCUUGGTT**		
**SLC2A1#2**	**Human**	**GUGCCAUACUCAUGACCAUTT**	**AUGGUCAUGAGUAUGGCACTT**		

**SLC2A3#1**	**Human**	**GCUCUUUCCAAUUUGGCUATT**	**UAGCCAAAUUGGAAAGAGCTT**
**SLC2A3#2**	**Human**	**GUAGCUAAGUCGGUUGAAATT**	**UUUCAACCGACUUAGCUACTT**
**Control**	**Human**	**UUCUCCGAACGUGUCACGUTT**	**ACGUGACACGUUCGGAGAATT**

Table S3: Supplementary Method Table

REAGENT or RESOURCE	SOURCE	IDENTIFIER
Antibodies		
Mouse monoclonal anti-GLTSCR1(H-10)	Santa	sc-515086
Rabbit monoclonal anti-LamiB1	Abcam	ab133741
Mouse monoclonal anti-GAPDH	Lianke	Cat#A85370844
Monoclonal ANTI-FLAG® M2 antibody	Sigma	F1804-50UG
Rabbit mAb anti-BRD4 (E2A7X)	CST	Cat#13440S for ChIP
Rabbit monoclonal anti-BRD4	Abcam	ab128874
Rabbit monoclonal anti-HA-tag	CST	Cat#3724S
Rabbit mAb anti-SSU72 (D3I2D)	CST	Cat#12816S
Rabbit monoclonal anti-SCP2	Abcam	Cat#ab140126
Rabbit monoclonal anti-CT1	Abcam	ab184703
Rabbit monoclonal anti-CDK9	Abcam	ab76320
Rabbit monoclonal anti-GST-tag	Abcam	ab19256
Rabbit monoclonal anti-His-tag	Abcam	ab213204
Rabbit monoclonal anti-KLF4	Abcam	ab215036
Rabbit monoclonal anti-SLC2A1	Abcam	ab115730
Rabbit monoclonal anti-SLC2A3	Abcam	ab191071

Rabbit monoclonal anti-RNA polymerase II (phospho S5)	Abcam	ab193467
Rabbit monoclonal anti-RNA polymerase II (phospho S2)	Abcam	ab193468
Rabbit monoclonal anti-HEXIM	Proteintech	Cat#66311-1-Ig
Rabbit monoclonal anti-NELFe	Abcam	ab170104
Rabbit Polyclonal anti-MLH1	Proteintech	Cat#11697-1-AP
Rabbit Polyclonal anti-MSH2	Proteintech	Cat#15520-1-AP
Mouse Monoclonal anti-MSH6	Proteintech	Cat#2E10B2
Mouse Monoclonal anti-PMS2	Proteintech	Cat#1G4E6
Rabbit mAb Flag tag antibody	CST	CAT#14793s
Mouse mAb Rpb1CTD (4H8)	CST	Cat#2629s
Purified Mouse Anti- BrdU	BD Pharmingen™	Cat#555627
Alexa® Fluor 488 Donkey anti-Mouse IgG (H+L)	ThermoFisher	Cat#A-21202
Alexa® Fluor 546 Goat anti-Rabbit IgG (H+L)	ThermoFisher	Cat#A-11010
IRDye® 800CW Goat-anti-Rabbit Antibody	LI-COR	Cat#926-32211
IRDye® 680CW Goat-anti-Mouse Antibody	LI-COR	Cat#926-68070
Bacterial		
DH5a	Vazyme	C502-02
BL21	Vazyme	C504-02
Biological Samples		
Patient samples	Run Run Shaw Hospital Zhejiang University	
Chemicals, Peptides, and Recombinant Proteins		

JQ-1	Targetmol	T2110
I-BET726	Targetmol	T6021
TBB	Targetmol	T2695-5MG
DRB	Sigma	Cat#D1916
TSA	Targetmol	T6270-5MG
G 418 disulfate salt	Sigma	G 5013
Puromycin	Sigma	Cat# P8833-10MG
D-luciferin	Goldbio	Cat# LUCNA (Luciferin, Potassium Salt)
Trizol	Invitrogen	Cat#15596018
Lipofectamine2000	Invitrogen	Cat#11668027
LipoD293	SignaGen	Cat # SL100668
GenMute siRNA Transfection Reagent	SignaGen	Cat # SL100568
Matrigel	BD Biosciences	Cat # 356234
Human Plasma Fibronectin Purified Protein	Millipore	FC010-1MG
Critical Commercial Assays		
Dual-Luciferase Reporter Assay Kit	Promega	E1910
GeneScan™ 500 LIZ™ Size Standard	ThermoFisher	Cat#4322682
BCA protein assay	ThermoFisher	Cat#23225
Glutathione Sepharose High Performance	GE Healthcare	Lot:10245887
TALON® Metal Affinity Resin	TAKARA	Cat#635503
ClonExpress-II One Step Cloning Kit	Vazyme	C112
SimpleChIP® Enzymatic Chromatin IP Kit	CST,	CAT#9003

CCK8	Bioshide	
SureBeads™ Protein A Magnetic Beads	Bio-RAD	Cat# 161-4013
Mut Express II Fast Mutagenesis Kit V2	Vazyme	C214
Anti-FLAG® M2 Magnetic Beads	Sigma	Cat#M8823
Deposited Data		
Raw and analyzed data	This paper	NCBI BioProject ID
Experimental Models: Cell Lines		
SW480	ATCC	
HCT116	ATCC	
SW620	ATCC	
HT29	ATCC	
Colo-205	ATCC	
HCT8	ATCC	
DLD1	ATCC	
HCT15	ATCC	
RKO	ATCC	
LOVO	ATCC	
HEK293	Chinese Academy of Sciences	
Experimental Models: Mice		
NOD–SCID–gamma	Shanghai SLAC Laboratory	www.slaccas.com
BALB/c nu/nu nude	Shanghai SLAC	www.slaccas.com

	Laboratory	
Oligonucleotides		
Primers for QPCR, see below	This paper	Table S2
siRNA SLC2A1-1	GenePharma	
siRNA SLC2A1-2	GenePharma	
siRNA SLC2A3-1	GenePharma	
siRNA SLC2A3-2	GenePharma	
siRNA Negative Control	GenePharma	
Recombinant DNA		
pLKO.1	This paper	
pGL3-basic	Gift from Pro. Shao Jimin	
pMD2G	This paper	
pRL-TK	Gift from Pro. Shao Jimin	
pGKV5	This paper	
pSPAX2	This paper	
pcDNA3.1(+)N-Flag	GenScript	
pcDNA3.1(+)N-Flag-GLTSCR1^{WT}-FL	GenScript	Cat#OHu07122C
pcDNA3.1(+)N-Flag-GLTSCR1$^{Del\text{-}FS}$-FL	GenScript	Cloned from Cat#OHu07122C
pcDNA3.1(+)N-Flag-GLTSCR1$^{In\text{-}FS}$-FL	GenScript	Cloned from Cat#OHu07122C

pcDNA3.1(+)N-Flag-GLTSCR1^{WT}(924-1561)	This paper	Cloned from pcDNA3.1(+) N-Flag-GLTSCR1^{WT}-FL
pcDNA3.1(+)N-Flag-GLTSCR1^{WT}(1161-1561)	This paper	Ditto
pcDNA3.1(+)N-Flag-GLTSCR1^{WT}(1361-1561)	This paper	Ditto
pcDNA3.1(+)C-HA-GLTSCR1^{WT}-FL	This paper	Ditto
pcDNA3.1(+)C-HA-GLTSCR1$^{Del\text{-}FS}$-FL	This paper	Ditto
pcDNA3.1(+)C-HA-GLTSCR1$^{In\text{-}FS}$-FL	This paper	Ditto
pcDNA3.1(+)C-HA-GLTSCR1$^{Del\text{-}FS}$+NLS	This paper	Ditto
pcDNA3.1(+)C-HA-GLTSCR1$^{In\text{-}FS}$+NLS	This paper	Ditto
p6344 pcDNA4-TO-HA-Brd4FL	Addgene	#31351
pcDNA3.1(+)C-HA-BDR4-FL	This paper	Cloned from Addgene plasmid #31351
pcDNA3.1(+)N-Flag-BDR4-FL	This paper	Ditto
pcDNA3.1(+)C-HA-BDR4(1-200)	This paper	Ditto
pcDNA3.1(+)C-HA-BDR4(170-348)	This paper	Ditto
pcDNA3.1(+)C-HA-BDR4(201-470)	This paper	Ditto
pcDNA3.1(+)C-HA-BDR4(1-470)	This paper	Ditto
pcDNA3.1(+)C-HA-BDR4(Δ292-299)	This paper	Ditto
pcDNA3.1(+)C-HA-BDR4(287-530)	This paper	Ditto
pcDNA3.1(+)C-HA-BDR4(530-722)	This paper	Ditto
pcDNA3.1(+)C-HA-BDR4(470-722)	This paper	Ditto
pcDNA3.1(+)C-HA-BDR4(1-722)	This paper	Ditto
pcDNA3.1(+)C-HA-BDR4(722-1362)	This paper	Ditto

pcDNA3.1(+)C-HA-BDR4(287-530)MUT#A	This paper	Ditto
pcDNA3.1(+)C-HA-BDR4(287-530)MUT#B	This paper	Ditto
pcDNA3.1(+)C-HA-BDR4(287-530)MUT#C	This paper	Ditto
pGEX-GST	Gift by Pro. Shao Jimin	
pGEX-GST-GLTSCR1 (1161-1361)	This paper	Cloned from pcDNA3.1(+) N-Flag-GLTSCR1WT-FL
pET-28a-His-BRD4 (1-470)	This paper	Cloned from Addgene plasmid #31351
pET-28a-His-BRD4 (285-530)	This paper	Ditto
Software and Algorithms		
GraphPadPRISM	Open source	https://www.graphpad.com
SPSS Statistics 20	Open source	
GeneMapper ID-X	Open source	https://www.thermofisher.com
ImageJ software	Open source	https://imagej.net/Welcome PMID 2293083
